# Blood diagnostic biomarkers for neurologic manifestations of long COVID

**DOI:** 10.1016/j.bbih.2025.101110

**Published:** 2025-09-25

**Authors:** A.S. Padhye, I.J. Koralnik, B.A. Hanson, L. Visvabharathy, R.K. DeLisle, G. Tachas

**Affiliations:** a Percheron Therapeutics Limited, Melbourne, Victoria, 3000, Australia; b Ken and Ruth Davee Department of Neurology, Feinberg School of Medicine, Northwestern University, Chicago, IL, 60611, USA; c Department of Surgery, University of Chicago, Illinois, 60637, USA; d Glysade LLC, Aurora, CO, 80016, USA

## Abstract

**Background:**

SARS-CoV-2 responsible for COVID-19 caused a global pandemic, with billions of infections, millions of deaths and ongoing manifestations post COVID-19. “Long Covid”, a Post-Acute Sequelae of COVID-19 (PASC), is an ongoing global healthcare problem, affecting all age groups, with many manifestations, and occurring despite vaccines and antivirals. Neurologic manifestations of PASC (Neuro-PASC) such as brain fog can last for years and are amongst the most debilitating and prevalent. There is a need for diagnostic tools and treatments.

**Methods:**

Plasma samples from 48 non-hospitalized PASC patients with diagnosed Neuro-PASC symptoms (NP), 20 convalescent control (CC) subjects, and 24 unvaccinated healthy control (HC) subjects, was used to generate data on over 7000 proteins using the SomaLogic® proteomics platform. ProViz® software was used to perform T-tests, U-Tests, ANOVA and Kruskalis-Wallis tests at a Bonferroni p < 0.05 and a Benjamini-Hochberg corrected False Discovery Rate <0.02, and box plots and knowhow used to identify diagnostic biomarkers and therapeutic targets.

**Results:**

C5a, TGFβ1, and Gliomedin, used together differentiated patients with Neuro-PASC from control subjects with 94 % sensitivity and 86 % specificity, a 90 % accuracy. Additional biomarkers, Gal3ST1, IFNλ1, and GHRH, improved accuracy to 94 %, and a combination of 5 more biomarkers, LFA-3, FASLG + Transgelin-1 and GPNMB + IGHG1, improved accuracy close to 100 %. These markers are suggestive of pathways involved in Neuro-PASC pathogenesis. A dozen partly overlying biomarkers were modulated to which there are FDA approved drugs.

**Conclusion:**

C5a, TGFβ1, Gliomedin expressed highly in serum could be developed as a diagnostic tool, and with clinical assessment used to personalize treatments with repurposed novel drugs.

## Highlights

• Long Covid currently affects 14 million Americans, with Neurologic manifestations of PASC (Neuro-PASC), cognitive impairment amongst the most debilitating, prevalent long-lasting manifestations
• A comprehensive proteomic approach assessing 7000 markers identified as few as 3 biomarkers differentiate patients with Neuro-PASC from control subjects with high sensitivity and specificity.
• C5a, TGFβ1, and Gliomedin, expressed highly in serum could be developed as a diagnostic tool with high 90 % accuracy, and in combination with up to 8 other markers, and clinical assessment used to personalize treatments with repurposed approved drugs.
• The high sensitivity and specificity biomarkers reveal altered neural, viral-pathogen, vascular-adipose, platelet, autoimmune (inflammatory, fibrosis, integrin, antigen presentation) pathways in Neuro-PASC.
• This novel approach may be used in investigations of mechanisms in other PASC and disease for the development of diagnostic and targeted therapeutic patient-centered interventions.

## 1 Introduction

Severe acute respiratory syndrome coronavirus 2 (SARS-CoV-2), the pathogen responsible for coronavirus disease 2019 (COVID-19) caused a global pandemic, with over 700 million people infected worldwide, and more than 7 million confirmed deaths recorded to mid-August 2022, 1 million just in the United States (Coronavirus disease). The COVID-19 pandemic total cost to the United States is estimated in 2020 to be over 16 trillion dollars (Cutler and Summers, 2020). Of the approximately 95 million people diagnosed with COVID-19 within the US by mid-August 2022, at just over 3 weeks post-infection, with COVID-19 lasting 4 weeks from onset of initial symptoms (Nalbandian et al., 2021), most are non-hospitalized for COVID-19 (Augustin et al., 2021) yet experience unresolved symptoms post-acute COVID-19 (Nalbandian et al., 2021)^,^ (O'Mahoney et al., 2023).

“Long COVID”, defined by WHO starting 3 months after initial COVID-19 infection with at least 2 months of persistent symptoms with no other explanation, and Post Acute Sequalae of SARS-CoV-2 infection (PASC), described in the US as “Post COVID” (Strong, 2022), including 4–12 weeks after initial infection subacute/ongoing COVID-19 phase, affects 22–38 % of people at 12 weeks, with 12–17 % having at least 3 symptoms in a chronic phase and estimated in July 2022 to have economic costs of $3.7 trillion dollars (The Economic Cost of Long, 2024).

The most reported Long COVID symptoms across non-hospitalized patients after 6 months are fatigue, post-exertional malaise, and cognitive dysfunction (Estiri et al., 2021; Davis et al., 2023). Cognitive symptoms are also reported in 26 % of subjects at 12 months and increased risk of cognitive impairment (brain fog), seizures, dementia, psychosis, and other neuro-cognitive conditions persistent at 2 years (Taquet et al., 2022). Within a group of 1700 people with PASC, 88 % reported some level of cognitive dysfunction, and 22 % were unable to work due to illness months after first diagnosed with COVID-19 (Davis et al., 2023). Persistent symptoms of brain fog, when measured using established memory tests identify loss of memory and attention, and are reported in 81 % of people with Neuro-PASC (Graham et al., 2021). Neuro-PASC is amongst the most debilitating and prevalent long-lasting manifestation in Long Covid and is in need of accurate diagnosis and characterization for personalized treatment.

While previous studies have identified various risk factors that may anticipate the onset of Long COVID at the time of diagnosis, including prior Epstein Barr Virus (EBV) viremia and Type 2 Diabetes, they have yet to provide diagnostic tools to accurately identify patients who are suffering from Long COVID (Su et al., 2022). Long COVID occurs with a changing incidence with different SARS-CoV-2 strains (Strong, 2022; Xie et al., 2024), a reduced risk with vaccination or antivirals (Bowe et al., 2023), but an increased risk with re-infections (Bowe et al., 2023), suggesting a major worldwide disability event is occurring (Strong, 2022; Bowe et al., 2023). The diagnosis and treatment of Long COVID is now a priority, with the National Institute of Health (NIH) committing additional funding to investigate its diagnosis and treatment alongside the US$1 billion it has already committed to its COVID-19 research efforts (NIH to bolster RECOVER Long, 2024).

Non pharmacological interventions reviewed in Frontera et al. in 2023 reported very low-to low-conditional support for improving pathological symptoms (Frontera et al., 2023). A recent narrative review of interventions on brain fog, processing speed and related cognitive outcomes including cognitive training, exercise and pharmacological intervention report on the scarce literature describing benefits in mental fatigue, such as a small study of 14 subjects, 6 self-reporting reduction in brain fog using antihistamines (Whitaker-Hardin et al., 2025). At present, hypothetical pathological mechanisms include viral persistence, neuro-invasion, neuroinflammation, chronic inflammation, immunological dysregulation, autoimmunity, coagulopathy, and vascular endothelial impairment and blood brain barrier (BBB) disruption (Paradiso et al., 2025). Whilst studies have look at transcriptomics of peripheral blood mononuclear cells relevant to some pathological mechanisms (Fineschi et al., 2025) and other studies have looked at a limited number of cytokines (Kwon et al., 2025; Robineau et al., 2025), there are still no established criteria for diagnosing long COVID and the persistent neurological manifestations (Saxena and Mautner, 2025).

Plasma samples from 48 non-hospitalized Long COVID patients diagnosed with neurological symptoms (such as cognitive difficulties) and fatigue, 20 convalescent control (CC) subjects, and 24 healthy control (HC) subjects, were used to generate data on over 7000 proteins using the SomaLogic® platform. Samples were statistically analyzed with Somalogic's® proprietary ProViz® software v1.1.0, using Bonferroni (BF)(Shi et al., 2012) and Benjamini-Hochberg (BH) False Discovery Rate (FDR) (Benjamini and Hochberg, 1995).

Plasma proteomics identified biomarkers that were significantly altered in Neuro-PASC patient samples, compared to CC and/or HC subjects, including biomarkers of peripheral nerve damage and of immune cell modulation.

Further unique analysis of Neuro-PASC biomarkers determined a small subset of 3 biomarkers C5a, Tgfβ1 and Gliomedin that when used in combination differentiated Neuro-PASC subjects from CC and HC subjects with high 94 % sensitivity (45/48) and 86 % specificity (38/44) providing a high 90 % accuracy.

These 3 primary biomarkers are highly abundant in serum, allowing them to be developed as a diagnostic tool using conventional methods to determine whether a patient is suffering from neurological Long COVID, and potentially how to treat them. When C5a and Tgfβ1 are highly expressed in Neuro-PASC patients they may benefit from treatment with existing drugs on the market and in late development targeting C5a and Tgfβ1, and the target and patient symptoms monitored. A group of 3 secondary biomarkers (Gal3ST1, IFN lambda-1, and GHRH) improved sensitivity, and specificity in the Neuro-PASC patients compared to HC subjects, and an additional tertiary group of 5 biomarkers improved Neuro-PASC specificity versus CC subjects. These diagnostic biomarkers, and other biomarkers differentially expressed to which there are FDA approved drugs open the way to understanding the mechanisms, and to the personalized treatment of Neuro-PASC subjects.

## 2 Materials and methods

### 2.1 Cohort

Patients who had been seen at the Neuro-COVID-19 clinic at the Northwestern Memorial hospital (NMH) were unvaccinated and first infected between September 2020 to June 2021. A total of 48 Neuro-PASC (NP) subjects who had tested PCR + for SARS-CoV-2 and reported ongoing neurological symptoms were sampled, 46 subjects from the NMH (group 1) and 2 patients recruited from the community not seen at any institute (group 2). The control group included 20 unvaccinated healthy CC subjects who had tested PCR + for SARS-CoV-2 but were all convalescent by 6 weeks and did not report any ongoing neurological symptoms (group 3), and 24 unvaccinated healthy control (HC) volunteers who tested PCR-for SARS-CoV-2 and showed no serum IgG anti-Spike RBD (group 4). The 20 subjects in the CC group and 24 subjects in the HC group were recruited through NMH and the community, and were screened and enrolled by members of the study research team. The NP and CC groups were age and sex-matched, as were the CC and HC groups and the NP and HC groups were sex-matched, and age related proteins in NP and HC groups excluded.

The study participants demographics are previously reported by Hanson et al. in Table 1 (Hanson et al., 2023). We were not able to compare comorbidities between NP, CC and HC participants, however, the frequency of comorbidities such as diabetes, obesity and pre-existing neurologic disease is generally low in non-hospitalized Neuro-PASC patients coming to our Northwestern Medicine (NM) Neuro-COVID-19 clinic (Giraldo et al., 2023). The NP study participant neurological symptoms of the 46 subjects from NMH who reported them are found in Hanson et al. (2023).

**Table 1:** T-Test and *U* Test Markers NP vs CC Bonferroni <0.05

T-test				
Protein Name	Uniprot ID	Gene Symbol	Bonferroni	Percent Change
SPARC	P09486	SPARC	0.043	−31 %

*U* test				
Protein Name	Uniprot ID	Gene Symbol	Bonferroni	Percent Change
Musculoskeletal embryonic-nuclear protein 1	Q8IVN3	MUSTN1	0.023	13 %
Galactosylceramide Sulfotransferase	Q99999	GAL3ST1	0.04	−22 %

Neurologic symptoms most frequently observed included brain fog (84.8 %) in 39/46 subjects, headache (78.3 %), dizziness (73.9 %), dysgeusia (71.7 %), and anosmia (69.6 %) Myalgia (58.7 %), Numbness/Tingling (50 %), Pain other than in the chest (50 %), Tinnitus (41.3 %) and Blurred Vision (34.8 %). Non-neurologic symptoms attributed to PASC most frequently reported were fatigue (89.1 %) in 41/46 subjects, depression and/or anxiety (82.6 %) in 38/46, and insomnia (65.2 %) in 30/46 subjects, shortness of Breath (60.9 %), Variations in heart rate/blood pressure, (47.8 %), GI symptoms (41.3 %) and Chest Pain (37 %).

The study was approved by the Northwestern University Institutional Review Board (STU00212583). All participants were enrolled after giving their written informed consent.

### 2.2 Plasma collection

Heparinized blood was collected on average 155–315 days post symptom onset. 30 mL of venous blood was collected in blood collection tubes with sodium heparin (BD Biosciences). Whole blood was separated into plasma and PBMC using Histopaque 1077 (Sigma) in 50 mL Leucosep blood separator tubes and spun at 1000×*g* for 18 min at RT. Plasma was collected and stored at −80 °C.

### 2.3 Proteomics data acquisition

Plasma samples were shipped with dry ice to SomaLogic® Inc. (Boulder, Colorado) where the SomaScan® assay was run to determine the relative abundance of proteins in the samples. SomaLogic® provided the data generated, expressed in log2 relative fluorescence units (RFU), enabled by protein-capture agents called “Slow Offrate Modified Aptamers” (SOMAmers®), quantified using hybridization to microarrays. These samples were compared against 5 pooled Calibrator Control samples, 3 Quality Control Samples (QC) replicates and 3 buffer (no protein) replicates on each 96-well plate, used to control for batch effects and to estimate the assays accuracy, precision, and buffer background levels. Samples were randomized across plates to minimize batch effects. The data was then subject to standardization performed by Somalogic® to mitigate any variance between runs. No differences in hybridization, intensity, assay, or systemic bias in raw assay data after aggregation were observed, and the data compared suitably to a global signal normalization reference. Aptamers were available to measure ∼7000 human protein analytes.

### 2.4 Data analysis and statistics

It was assumed control CC (group 3, n = 20) and control HC (group 4, n = 24) would not be significantly different for analytes in the SomaScan® dataset and that 44 control subjects compared to 48 NP (group 1 + 2, n = 48) subjects would be 80 % powered to see an effect size of 0.6; and subsequently showed no biomarkers differed between controls.

NP Samples, (group 1 + 2, n = 48) were first compared to CC (group 3, n = 20) or HC (group 4, n = 24) using a two-tailed T-test or *U* test and a Bonferroni (BF) p value of <0.05 for human analytes and then using a Benjamini-Hochberg (BH) corrected False Discovery Rate (FDR) at <0.05, <0.02, and <0.01.

T-test and *U* test BF and BH corrected FDR <0.05 identified no proteins differentially expressed between the CC (group 3, n = 20) and HC (group 4, n = 24) subjects. T-test and U-tests were thus conducted comparing NP samples (group 1 + 2, n = 48) with the combined CC and HC group samples (n = 44), using BF < 0.05 for human analytes and an FDR of <0.02 and < 0.01.

The NP, CC, and HC groups were also assessed by parametric ANOVA and non-parametric Kruskal-Wallis (KW) tests, a BF of <0.05 for human analytes identifying 31 and 9 targets respectively, a BH corrected FDR of <0.01 identifying 149 and 118 targets respectively and a BH corrected FDR of <0.02 identifying 314 and 284 targets respectively.

In the above, the BF value for significance was set at <0.052 for the SomaScan® analytes, corresponding to the BF < 0.05 for human analytes in the SomaScan® test.

### 2.5 Pathway analysis

A pathway analysis (Genetrails3) was performed on the 314 protein markers identified in the ANOVA (NP v CC or HC) BH corrected FDR <0.02.

## 3 Results

### 3.1 NP subjects compared to CC subjects using a T-test and *U* test Bonferroni <0.05 analysis identified 3 biomarkers

Using parametric T-test, only 1 protein, SPARC was identified as being significantly lower in NP subjects (median reduced 31 %) compared to CC subjects with a BF < 0.043 (Table 1). Within the brain, SPARC is expressed in blood vessels, its expression is acutely depressed in adolescents post-concussion (Miller et al., 2021), though higher in vessels close to injury, and deficiency can lead to a reduction in synaptic plasticity during the development of the nervous system (Jones et al., 2011). SPARC is also involved in remodeling of the extracellular matrix and recruitment of antigen-specific T-cells into the brain following infection (McGovern et al., 2021).

Using a Non-parametric *U* Test, only 2 proteins, MUSTN1, a skeletal muscle and smooth muscle protein, and GAL3ST1, an enzyme that synthesizes sulfatide in myelin sheath neurons expressed in brain oligodendrocytes and intestinal enterocytes, were identified as expressed significantly differently between the NP and CC groups. The median MUSTN1 level was 13 % higher in NP subjects while GAL3ST1 was 22 % lower in NP subjects (Table 1) (GAL3ST1 protein expression summary, MUSTN1 protein expression summary).

### 3.2 NP subjects compared to HC subjects using a T-test and *U* test Bonferroni <0.05 analysis identified 30 biomarkers

When NP subjects were compared to HC subjects using a Parametric T-test, 30 targets were identified as differentially expressed in NP versus HC subjects by BF at <0.05. 20 of these 30 targets were shared when NP subjects were compared to HC subjects using a non-parametric *U* test (Table 2a) and 10 were unique to the T-test (Table 2b).

**Table 2a:** Targets Shared in T-test and *U* test NP vs HC subjects Bonferroni <0.05

T-test					*U* Test
Protein Name	Uniprot ID	Gene Symbol	Bonferroni	Percent Change	Bonferroni
Glycerol-3-phosphate dehydrogenase [NAD(+)], cytoplasmic	P21695	GPD1	0.00094	114 %	0.034
Fatty acid-binding protein, adipocyte	P15090	FABP4	0.0082	100 %	0.0087
Ribonuclease pancreatic	P07998	RNASE1	0.0064	69 %	0.015
IGF-like family receptor 1	Q9H665	IGFLR1	0.0024	60 %	0.0052
Glycosyltransferase-like protein LARGE1	O95461	LARGE1	0.049	54 %	0.015
Macrophage scavenger receptor types I and II:Extracellular domain	P21757	MSR1	0.013	52 %	0.021
Triggering receptor expressed on myeloid cells 2	Q9NZC2	TREM2	0.0022	45 %	0.007
Cathepsin S	P25774	CTSS	0.0024	44 %	0.034
Protein FAM84B	Q96KN1	LRATD2	0.044	43 %	0.034
CUB domain-containing protein 1	Q9H5V8	CDCP1	0.0044	35 %	0.038
Somatoliberin	P01286	GHRH	0.00035	34 %	0.0056
Complement C1q tumor necrosis factor-related protein 3	Q9BXJ4	C1QTNF3	0.0026	33 %	0.021
Tumor necrosis factor receptor superfamily member 1B	P20333	TNFRSF1B	0.034	33 %	0.012
Carbohydrate sulfotransferase 11	Q9NPF2	CHST11	0.0081	32 %	0.0094
Plexin-B2	O15031	PLXNB2	0.011	31 %	0.034
Cadherin-23	Q9H251	CDH23	0.031	−17 %	0.024
Asparagine synthetase	P08243	ASNS	3.60E-05	−18 %	7.30E-05
Ras-related protein Rab-35	Q15286	RAB35	8.60E-05	−19 %	8.00E-05
Transmembrane gamma-carboxyglutamic acid protein 1:Cytoplasmic domain	O14668	PRRG1	0.011	−21 %	0.035
BTB/POZ domain-containing adapter for CUL3-mediated RhoA degradation protein 1	Q8WZ19	KCTD13	0.00011	−30 %	2.00E-04

**Table 2b:** T-test Unique Targets NP vs HC Bonferroni <0.05

Protein Name	Uniprot ID	Gene Symbol	Bonferroni	Percent Change	
39S ribosomal protein L33, mitochondrial	O75394	MRPL33	0.036	248 %	
C-type lectin domain family 12 member A	Q5QGZ9	CLEC12A	0.0081	130 %	
SPARC-like protein 1	Q14515	SPARCL1	0.02	114 %	
Macrophage scavenger receptor types I and II:Extracellular domain	P21757	MSR1	0.013	52 %	
Secreted frizzled-related protein 1	Q8N474	SFRP1	0.019	46 %	
Tumor necrosis factor ligand superfamily member 6, soluble form	P48023	FASLG	0.03	46 %	
Microfibrillar-associated protein 2	P55001	MFAP2	0.012	38 %	
Oxidized Protein deglycase DJ-1	Q99497	PARK7	0.014	−14 %	
Cytochrome *b* reductase 1	Q53TN4	CYBRD1	0.049	−38 %	
Protein phosphatase 1F	P49593	PPM1F	0.045	−59 %	
					

When NP subjects were compared to HC subjects using a non-parametric *U* test, 29 targets were identified as differentially expressed in NP versus HC subjects by BF at <0.05, with 9 being unique to the *U* Test (Table 2c).

**Table 2c:** *U* test Unique Targets NP vs HC Bonferroni <0.05

Protein Name	Uniprot ID	Gene Symbol	Bonferroni	Percent Change
Promotilin	P12872	MLN	0.036	130 %
Synaptotagmin-6	Q5T7P8	SYT6	0.029	45 %
Tumor necrosis factor receptor superfamily member 1A	P19438	TNFRSF1A	0.034	40 %
Transcription initiation factor TFIID subunit 10	Q12962	TAF10	0.0065	−10 %
Tyrosine-protein kinase ZAP-70	P43403	ZAP70	0.0011	−12 %
Alpha-2-HS-glycoprotein	P02765	AHSG	0.044	−16 %
Regulatory factor X-associated protein	O00287	RFXAP	0.05	−18 %
Acylpyruvase FAHD1, mitochondrial	Q6P587	FAHD1	0.038	−49 %
Tetratricopeptide repeat protein 9A	Q92623	TTC9	0.041	−50 %

A total of 39 targets were identified using parametric and non-parametric tests, with 20 targets shared between both groups.

There were more than a 10-fold greater number of total targets identified in the NP versus HC groups (Table 2a–c) compared to the NP versus CC groups (Table 1), suggesting the CC group is closer to the NP subjects, though with 24 HC versus 20 CC subjects a greater number of subjects may have also contributed to higher numbers identified versus HC.

### 3.3 CC subjects compared to HC subjects using a T-test and *U* test Bonferroni <0.05: No biomarkers differed statistically significantly by BF < 0.05

When the HC and CC subjects were compared using a T-Test or a *U* test, there were no statistically significant changes in any plasma protein via BF < 0.05 for human analytes. All proteins identified had a BF > 0.77 and >0.49 in the T-test and *U* test respectively, and FDR >0.52 and >0.29 respectively suggesting HC and CC samples were relatively similar at the biomarker level.

### 3.4 NP subjects compared to combined CC + HC subjects, using a T-test and *U* test Bonferroni of <0.05 identified an additional 13 biomarkers

As there were no statistically significant differentially expressed protein changes between the CC and HC groups, the 48 NP subjects were compared to 44 subjects from the combined CC (n = 20) and HC (n = 24) control groups. Using a BF of <0.05 for human analytes identified 45 targets differentially expressed with the T-test and 45 with the *U* test, with 29 shared between the two different tests (Table 3a) and 16 unique for each group (Table 3b and c).

**Table 3a:** Targets Shared between T-test and *U* test NP vs Controls (CC + HC) Bonferroni <0.05

T-test					*U* Test
Protein Name	Uniprot ID	Gene Symbol	Bonferroni	Percent Change	Bonferroni
Fatty acid-binding protein, adipocyte	P15090	FABP4	0.015	77 %	0.013
Cellular retinoic acid-binding protein 2	P29373	CRABP2	0.0029	69 %	0.015
Ribonuclease pancreatic	P07998	RNASE1	8.50E-03	67 %	0.017
Four-jointed box protein 1	Q86VR8	FJX1	0.0083	55 %	0.021
Macrophage scavenger receptor types I and II:Extracellular domain	P21757	MSR1	0.0053	51 %	0.0063
Transgelin (9756-6)	Q01995	TAGLN	0.0097	51 %	0.014
Prostaglandin-H2 D-isomerase	P41222	PTGDS	0.0055	45 %	0.018
Glycosyltransferase-like protein LARGE1	O95461	LARGE1	9.60E-03	43 %	0.025
CUB domain-containing protein 1	Q9H5V8	CDCP1	0.007	41 %	0.0087
IGF-like family receptor 1	Q9H665	IGFLR1	0.0048	40 %	0.0058
Triggering receptor expressed on myeloid cells 2	Q9NZC2	TREM2	0.014	39 %	0.021
Tumor necrosis factor receptor superfamily member 1A	P19438	TNFRSF1A	0.0068	38 %	0.0073
Gliomedin	Q6ZMI3	GLDN	0.0019	34 %	0.0018
Protein FAM84B	Q96KN1	LRATD2	0.041	32 %	0.0055
Ribonuclease T2	O00584	RNASET2	0.019	28 %	0.041
Carbohydrate sulfotransferase 11	Q9NPF2	CHST11	0.0031	27 %	0.0058
Tumor necrosis factor receptor superfamily member 1B	P20333	TNFRSF1B	0.011	27 %	0.0055
Plexin-B2	O15031	PLXNB2	0.0025	23 %	0.017
Transmembrane glycoprotein NMB:Extracellular domain	Q14956	GPNMB	0.022	21 %	0.045
Plexin domain-containing protein 2:Extracellular domain	Q6UX71	PLXDC2	0.0079	18 %	0.026
N-acetylglucosamine-1-phosphotransferase subunit gamma	Q9UJJ9	GNPTG	0.0076	12 %	0.015
Tumor necrosis factor receptor superfamily member 10B	O14763	TNFRSF10B	0.0062	11 %	0.01
Guanylate-binding protein 5	Q96PP8	GBP5	0.0085	−10 %	0.039
Asparagine synthetase	P08243	ASNS	0.00064	−14 %	0.00086
Fragile X mental retardation protein 1	Q06787	FMR1	0.033	−15 %	0.046
Ras-related protein Rab-35	Q15286	RAB35	0.0063	−16 %	0.0073
NADH dehydrogenase [ubiquinone] 1 alpha subcomplex subunit 2	O43678	NDUFA2	0.0097	−16 %	0.011
Apolipoprotein D	P05090	APOD	0.015	−17 %	0.041
Palmitoyl-protein thioesterase 1	P50897	PPT1	0.016	−18 %	0.008

**Table 3b:** T-test Unique Targets NP vs Controls (CC + HC) Bonferroni <0.05

Protein Name	Uniprot ID	Gene Symbol	Bonferroni	Percent Change
Carbohydrate sulfotransferase 15	Q7LFX5	CHST15	0.012	114 %
Glycerol-3-phosphate dehydrogenase [NAD(+)], cytoplasmic	P21695	GPD1	0.04	78 %
Glycolipid transfer protein domain-containing protein 2	A6NH11	GLTPD2	0.044	61 %
Thrombospondin-4	P35443	THBS4	0.026	52 %
Ephrin type-A receptor 6	Q9UF33	EPHA6	0.025	47 %
SLIT and NTRK-like protein 2	Q9H156	SLITRK2	0.0099	46 %
Transgelin (15640-54)	Q01995	TAGLN	0.023	46 %
Frizzled-7	O75084	FZD7	0.032	42 %
Alpha-1,6-mannosylglycoprotein 6-beta-N-acetylglucosaminyltransferase A	Q09328	MGAT5	0.02	34 %
Regulator of G-protein signaling 4	P49798	RGS4	0.014	30 %
Ephrin type-B receptor 2	P29323	EPHB2	0.024	30 %
Aldehyde dehydrogenase X, mitochondrial	P30837	ALDH1B1	0.025	−12 %
Oxidized Protein deglycase DJ-1	Q99497	PARK7	0.012	−14 %
BTB/POZ domain-containing adapter for CUL3-mediated RhoA degradation protein 1	Q8WZ19	KCTD13	0.036	−28 %
Cytochrome *b* reductase 1	Q53TN4	CYBRD1	0.023	−35 %
Alanyl-tRNA editing protein Aarsd1	Q9BTE6	AARSD1	0.024	−36 %

**Table 3c:** *U* test Unique Targets NP vs Controls (CC + HC) Bonferroni <0.05

Protein Name	Uniprot ID	Gene Symbol	Bonferroni	Percent Change
EH domain-containing protein 2	Q9NZN4	EHD2	0.017	42 %
Leukocyte cell-derived chemotaxin-2	O14960	LECT2	0.016	39 %
Complement C1q tumor necrosis factor-related protein 3	Q9BXJ4	C1QTNF3	0.045	30 %
Ectonucleoside triphosphate diphosphohydrolase 1	P49961	ENTPD1	0.026	26 %
DNA damage-inducible transcript 3 protein	P35638	DDIT3	0.023	21 %
Multiple PDZ domain protein	O75970	MPDZ	0.035	15 %
Musculoskeletal embryonic nuclear protein 1	Q8IVN3	MUSTN1	0.015	13 %
Discoidin domain-containing receptor 2	Q16832	DDR2	0.013	13 %
Cyclin-dependent kinase 8:Cyclin-C complex	P49336|P24863	CDK8|CCNC	0.032	−12 %
Zinc finger and BTB domain-containing protein 7A	O95365	ZBTB7A	0.016	−14 %
B-cell receptor CD22	P20273	CD22	0.016	−14 %
Protein phosphatase 1G	O15355	PPM1G	0.026	−15 %
40S ribosomal protein S12	P25398	RPS12	0.021	−20 %
Galactosylceramide sulfotransferase	Q99999	GAL3ST1	0.021	−23 %
Acylpyruvase FAHD1, mitochondrial	Q6P587	FAHD1	0.027	−37 %
Tetratricopeptide repeat protein 9A	Q92623	TTC9	0.014	−50 %

The MUSTN1 and GAL3ST1, identified by BF < 0.05 in NP versus CC *U* test were again identified in the NP vs the combined CC and HC groups *U* test data at BF p = 0.015 and p = 0.021 respectively, consistent with the finding in the NP vs CC group *U* test.

SPARC, which was previously found to be differentially expressed by BF T-test in NP vs CC groups (Table 1), was only significantly different by non-parametric *U* test analysis when comparing NP vs combined CC and HC using BH adjusted FDR (p = 0.004) and so does not appear in Table 3a, Table 3b, Table 3c BF data.

### 3.5 NP subjects versus CC T-test and *U* test BH FDR analysis to <0.05

Using BH corrected FDR <0.05, 25 and 53 proteins were identified with the T-test and *U* test respectively, comparing the 48 NP group samples to the 20 CC samples (Supplementary table 1). In the 53 *U* test proteins, 29 biomarkers were elevated in the plasma of NP subjects compared to CC subjects with 3 biomarkers having medians increased >50 %. Of the 24 remaining biomarkers that decreased, 3 decreased by > 50 %. The T-test and *U* test results detect identical markers at both % ends of the spectrum, with the same 3 biomarkers showing the greatest increase and 2 of the 3 proteins with the greatest decrease in the *U* test appearing at the bottom of the T-test.

At a BH FDR <0.075, there were 65 biomarkers that showed a significant change when the 48 NP subjects were compared to 20 CC subjects using a T-test and 344 biomarkers using a *U* test (Supplementary table 1). Of the 65 T-test biomarkers 45 biomarkers were elevated in NP versus CC subjects, 3 biomarkers with an increase in means of >50 %, and 20 biomarkers showed a decrease, 4 with a decrease in means of >50 %.

The non-parametric *U* test identified more than 5-fold differentially expressed targets than the parametric T-test at FDR <0.075, and more than 2X more targets at FDR <0.05.

### 3.6 NP subjects versus HC T-test and *U* test BH FDR analysis at FDR <0.01

Using BH corrected FDR <0.01, 184 and 213 proteins were identified as differentially expressed with the T-test and *U* test respectively, comparing the 48 NP group samples to 24 HC samples (Supplementary table 2).

### 3.7 NP subjects versus combined CC + HC T-test and *U* test FDR analysis at FDR <0.01

Using BH corrected FDR <0.01, there were 440 biomarkers significantly differentiated in the NP versus combined control CC + HC groups via the T-test and 493 via the *U* test.

### 3.8 NP compared to the CC and HC subjects using ANOVA and KW Bonferroni of <0.05

Using Bonferroni of <0.05 for human analytes, the ANOVA and KW tests comparing NP group samples to each of the CC and HC samples, 31 and 9 biomarkers were identified respectively. Of the 9 biomarkers in the KW test, 7 were also identified in the ANOVA, leaving 2 additional biomarkers uniquely identified from the KW test.

The 33-protein marker changes in NP subjects compared to CC and HC using an ANOVA and KW are in (Table 4).

**Table 4:** ANOVA Results comparing the NP, CC, HC groups (Bonferroni <0.05).

Protein Name	UniProt ID	Gene Symbol	Bonferroni	Percentage change
Carbohydrate sulfotransferase 15	Q7LFX5	CHST15	0.033	56 %
Glycerol-3-phosphate dehydrogenase [NAD(+)], cytoplasmic	P21695	GPD1	0.051	53 %
Fatty acid-binding protein, adipocyte	P15090	FABP4	0.041	50 %
Cellular retinoic acid-binding protein 2	P29373	CRABP2	0.01	41 %
Ribonuclease pancreatic	P07998	RNASE1	0.021	41 %
IGF-like family receptor 1	Q9H665	IGFLR1	0.012	38 %
Four-jointed box protein 1	Q86VR8	FJX1	0.039	38 %
Glycosyltransferase-like protein LARGE1	O95461	LARGE1	0.012	35 %
Macrophage scavenger receptor types I and II:Extracellular domain	P21757	MSR1	0.018	34 %
SLIT and NTRK-like protein 2	Q9H156	SLITRK2	0.038	34 %
CUB domain-containing protein 1	Q9H5V8	CDCP1	0.042	32 %
Cathepsin S	P25774	CTSS	0.018	31 %
Prostaglandin-H2 D-isomerase	P41222	PTGDS	0.021	31 %
Synaptotagmin-6	Q5T7P8	SYT6	0.028	31 %
Triggering receptor expressed on myeloid cells 2	Q9NZC2	TREM2	0.035	31 %
Gliomedin	Q6ZMI3	GLDN	0.015	29 %
Tumor necrosis factor receptor superfamily member 1A	P19438	TNFRSF1A	0.036	29 %
Tumor necrosis factor receptor superfamily member 1B	P20333	TNFRSF1B	0.04	25 %
Plexin-B2	O15031	PLXNB2	0.0052	24 %
Carbohydrate sulfotransferase 11	Q9NPF2	CHST11	0.0093	24 %
N-acetylglucosamine-1-phosphotransferase subunit gamma	Q9UJJ9	GNPTG	0.043	12 %
Guanylate-binding protein 5	Q96PP8	GBP5	0.025	−12 %
Oxidized Protein deglycase DJ-1	Q99497	PARK7	0.047	−18 %
Asparagine synthetase	P08243	ASNS	0.00048	−21 %
Cadherin-23	Q9H251	CDH23	0.051	−21 %
Ras-related protein Rab-35	Q15286	RAB35	0.00019	−23 %
Apolipoprotein D	P05090	APOD	0.051	−25 %
Transmembrane gamma-carboxyglutamic acid protein 1:Cytoplasmic domain	O14668	PRRG1	0.024	−27 %
BTB/POZ domain-containing adapter for CUL3-mediated RhoA degradation protein 1	Q8WZ19	KCTD13	0.0027	−43 %
Cytochrome *b* reductase 1	Q53TN4	CYBRD1	0.02	−62 %
Alanyl-tRNA editing protein Aarsd1	Q9BTE6	AARSD1	0.037	−83 %

BF value for significance was set at <0.052 for the SomaScan® analytes, corresponding to the BF < 0.05 for human analytes in the SomaScan® test.

The 2 additional proteins identified in the KW non-parametric analysis were, Somatoliberin (GHRH) up by a median 25 % in NP vs CC or HC, and Palmitoyl Protein thioesterase 1 (PPT1) down by a median 22 % in NP vs CC or HC. For GHRH NP versus HC was up 34 % (BF = 0.0056) and for PPT1 NP was down versus HC (−17 %, FDR = 0.0047) and versus CC (−18 % FDR = 0.039).

### 3.9 NP compared to the CC and HC subjects using ANOVA and KW BH FDR <0.02

Using BH corrected FDR <0.0005 for human analytes, 2 biomarkers (RAB35, p = 0.00019 and ASNS, p = 0.00048) and 0 biomarkers were identified with the ANOVA and KW tests respectively comparing NP group samples, to CC group 3 or HC group 4 samples.

At a BH corrected FDR <0.01, 149 biomarkers showed a significant change when comparing NP subjects to HC or CC subjects with ANOVA and 118 with KW (Supplementary Table 3).

At a BH corrected FDR <0.02, 314 biomarkers showed a significant change in HC or CC subjects versus NP subjects via ANOVA and 284 via KW (Supplementary Table 4).

### 3.10 Pathway analysis of ANOVA BH FDR <0.02

The data from 314 biomarkers identified as differentially expressed in the ANOVA BH FDR <0.02 were assessed by pathway analysis using Genetrails3, with KEGG, Reactome and GO as the databases. Biomarkers identified involved platelet degranulation-clotting, viral mRNA translation, TGF-β signaling, activation of nfkappa B in B cells, antigen processing, regulation of complement, integrin cell surface interactions including with the vascular wall, IL-1 signaling and potentially related TLR 2/4, Traf6, white adipocyte differentiation, Circadian clock, glycogen, iron pathways and others as indicated in Table 5. Circadian clock may be related to insomnia in the non-neurological conditions.

**Table 5:** Summary of Pathway Analysis of targets of interest at ANOVA BH FDR <0.02

Name	Number of hits	Expected score	Adjusted p-value
Platelet degranulation	10	0.132031	2.77E-013
Viral mRNA Translation	5	0.140388	2.08E-005
TGF-beta receptor signaling in EMT (epithelial to mesenchymal transition)	3	0.0267405	8.45E-005
Activation of NF-kappaB in B cells	4	0.110305	1.35E-004
Transcriptional regulation of white adipocyte differentiation	4	0.132031	2.59E-004
Downregulation of TGF-beta receptor signaling	3	0.0434534	2.81E-004
Integrin cell surface interactions	4	0.142059	3.29E-004
Assembly Of The HIV Virion	2	0.00835642	5.93E-004
Antigen processing: Ubiquitination & Proteasome degradation	5	0.371025	7.99E-004
Circadian Clock	3	0.0802216	1.41E-003
RNA Polymerase I Chain Elongation	3	0.0952631	1.97E-003
Apoptotic cleavage of cell adhesion proteins	2	0.0183841	2.14E-003
IRAK4 deficiency (TLR2/4)	2	0.0183841	2.14E-003
MyD88 deficiency (TLR2/4)	2	0.0183841	2.14E-003
IRAK1 recruits IKK complex upon TLR7/8 or 9 stimulation	2	0.023398	3.07E-003
Glycogen synthesis	2	0.0250692	3.27E-003
Iron uptake and transport	2	0.0250692	3.27E-003
TRAF6 mediated IRF7 activation in TLR7/8 or 9 signaling	2	0.0250692	3.27E-003
RNA Polymerase III Chain Elongation	2	0.0300831	4.29E-003
Toll Like Receptor 4 (TLR4) Cascade	2	0.0300831	4.29E-003
Intrinsic Pathway of Fibrin Clot Formation	2	0.0367682	5.81E-003
Abortive elongation of HIV-1 transcript in the absence of Tat	2	0.0384395	6.09E-003
Regulation of Complement cascade	2	0.0401108	6.48E-003
Interleukin-1 signaling	2	0.0584949	1.18E-002
NCAM1 interactions	2	0.0701939	1.58E-002
Cell surface interactions at the vascular wall	2	0.0902493	2.40E-002

Among the statistically significantly altered proteins between Neuro-PASC subjects and CC and/or HC, the majority are involved in platelet, viral, immunological pathways of B-cell which can be the source of IL1 inflammation, TGF-β signaling, and complement. The plasma proteins identified as differentially expressed which can also be used for diagnostic tools in various combination include TGF- β and C5a for sensitivity and IGHG1 and adhesion molecule LFA-3 for specificity, which serve as targets for potential monitoring and therapeutic intervention (Table 6).

**Table 6:** Potential Therapeutic Targets Identified with ANOVA statistical analysis of NP vs Control subject Exemplary Biomarker Test Combinations for Diagnosis of Neuro-PASC.

Protein Name	FDR	% change	Known Drug/Modulator
**Increased levels with Neuro-PASC**			
Complement C5b-C6 complex^a^	0.007	28 %	Ravulizumab
Lymphocyte function-associated antigen 3 CD58^a^	0.01	15 %	Alefacept
Transforming growth factor beta-1 (TGF-β1)^a^	0.011	21 %	Pirfenidone
Vascular endothelial growth factor D (VEGF-D)^a^	0.014	31 %	OPT-302
Myeloid cell surface antigen CD33^a^	0.014	23 %	Gemtuzumab
Alcohol dehydrogenase 1B^a^	0.01	22 %	Fonepizole (CAS 7554-65-6)
Tumor necrosis factor receptor superfamily (TNFRSF) member 1A^a^	0.0016	29 %	Atrosimab
Tumour Necrosis Factor Receptor Superfamily (TNFRSF)10b^a^	0.0016	11 %	Conatumumab and Lexatumumab
Cathepsin S^a^	0.018	31 %	Petesicatib
TLR4: Lymphocyte antigen 96 complex^a^	0.0087	32 %	Resatorvid or Eritoran
Complement component 5a (C5a)^a^	0.016	50 %	siRNA against C5a
Growth Hormone Releasing Hormone (GHRH)^a^/b	0.0028	25 %	MR 409
**Decreased levels with Neuro-PASC**			
BCL2 like 1 (Bcl2L1)^b^	0.0063	−21 %	Navitoclax (ABT-263) or Obatoclax
B-cell receptor CD22^b^	0.0071	−22 %	NA
Antithrombin-III^b^	0.011	−25 %	Heparin
Protein farnesyltransferase^b^	0.015	−34 %	NA
Amyloid A4 protein^b^	0.017	−48 %	NA
Thyroid peroxidase^b^	0.018	−15 %	NA
IGHG1^b^	0.0088	−29 %	IgG from COVID convalescent patient plasma.
Palmitoyl protein thioesterase^b^	0.0027	−22 %	NA

∗/∗∗GHRH, if inducing problematic TH17 inflammation may be targeted with an antagonist to GHRH, or to growth hormone action, like pegvisomant. GHRH if high GHRH is beneficial may be modulated via agonist Growth Hormone (GH), or Insulin like growth factor 1 (IGF-I).

a Exemplary Neuro-PASC Therapeutic Targets to be inhibited.

b Exemplary Neuro-PASC Therapeutic Targets to be Activated/Modulated.

### 3.11 Therapeutic targets

The 314 proteins identified to be differentially expressed in NP subjects using ANOVA with a BH corrected FDR <0.02 were reviewed to determine which had drugs that may potentially be used as treatments (Zhou et al., 2024). Of these targets, 12 had FDA approved drugs on the market as of 2023, while an additional 19 had drugs that were in either Phase II or III trials, with 2 and 3 of these drugs in Phase II and III trials respectively for SARS-CoV-2 (Table 6).

Some of the proteins may be one of the underlying causes of the aberrant response that persists into the post-acute phase and through therapeutic intervention may be altered in levels or targeted to alleviate the condition.

### 3.12 Diagnostic Algorithm

The SomaScan® proteomics assay and ProViz® software enabled detection of biomarkers significantly differentiated via the T-test, *U* test, using BF, and BH adjusted FDR and corresponding ANOVA and KW tests and the statistically significantly modulated targets evaluated further to identify potential diagnostic targets.

Using biological knowhow algorithms (Tachas and Padhye) and box plots it was possible to identify biomarkers for the diagnosis of NP subjects compared to CC and HC controls and inform on potential personalized treatments.

**Fig. 1:**
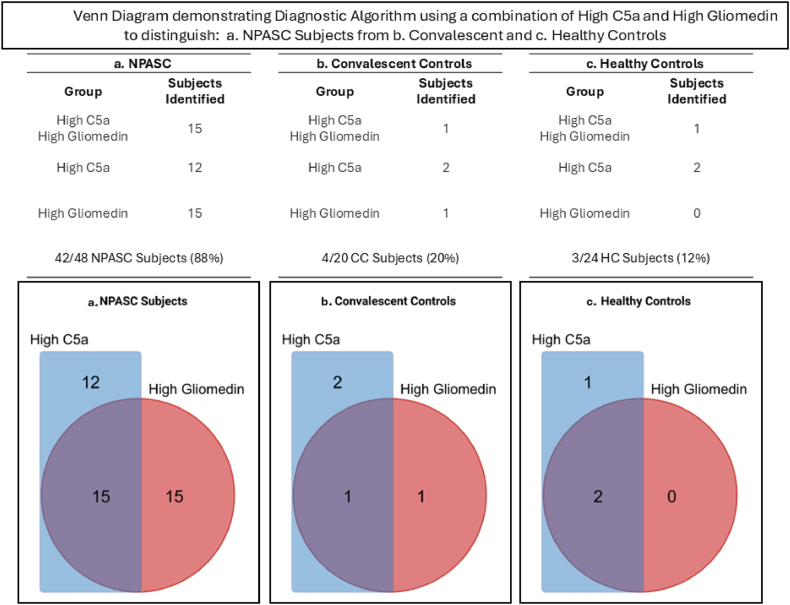
Venn Diagram demonstrating Diagnostic Algorithm using a combination of High C5a and High Gliomedin to distinguish: a. NPASC Subjects from b. Convalescent and c. Healthy Controls.

Measuring C5a and/or Gliomedin levels higher than a threshold level in controls identified NP subjects with approximately 88 % sensitivity (42/48) (Fig. 1a) and provided 80 % specificity versus CC subjects (16/20), and 88 % versus HC subjects (20/24) (Fig. 1b and c). This left 4/20 CC and 3/23 HC subjects that were not distinguished by that approach. 1 of these 3 HC subjects in Fig. 1c had extreme C5a levels, 5 times the upper end of HC, and twice the upper level of NP subjects, so this extreme outlier could be distinguished via extremely high C5a, providing 38/44, 86 % specificity versus the HC and CC controls using 2 biomarkers. Alternatively, excluding this 1 HC subject as an extreme outlier high C5a, would similarly provide 86 % specificity (37/43).

**Fig. 2:**
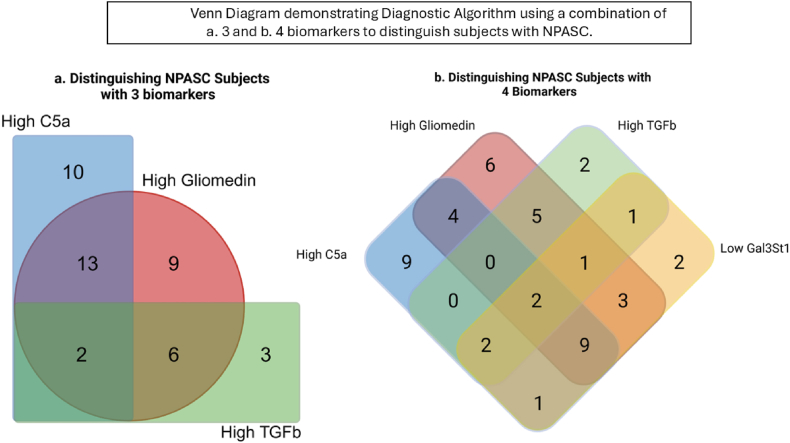
Venn Diagram demonstrating Diagnostic Algorithm using a combination of a. 3 and b. 4 biomarkers to distinguish subjects with NPASC.

Adding a third marker high TGF-β1, and measuring for high C5a and/or high Gliomedin and/or high TGF-β1 levels, TGF-β1 higher than a threshold level in controls identified 3 more of the NP subjects (45/48) providing approximately 94 % sensitivity Fig. 2a, and as described above with (extremely) high C5a and/or Gliomedin, 86 % specificity (38/44). Thus, the 3 primary biomarkers of high C5a, Gliomedin and TGF-β1, had 90 % accuracy versus combined controls when used in the described manner.

Adding a fourth marker, low Galactosylceramide sulfotransferase (GAL3ST1), identified, another 2 NP subjects, 47/48 NP subjects overall providing 98 % sensitivity (Fig. 2b–Table 7 above the first broken line). The 45/48 NP subjects in Fig. 2a that tested positive for High C5a, and/or High Gliomedin and/or high TGF-β1 all had normal levels of GAL3ST1 while 2 different NP subjects had reduced levels of GAL3ST1 below a threshold. Using the first 3 biomarkers and GAL3ST1, the test would thus provide 98 % sensitivity (47/48) as shown in Fig. 2b–Table 7; and 86 % average specificity versus the CC + HC controls (via C5a and Gliomedin) and thus 92 % accuracy (Table 7).

**Table 7:** – Exemplary biomarker test combinations for diagnosis of Neuro-PASC.

				Changes vs earlier row
Biomarker Combination (accuracy)	Average Sensitivity (%)	Average Specificity (%)	CC Specificity (%)	HC Specificity (%)
**2 markers**: **(87 % accuracy)** C5a (high) and/or Gliomedin (high)	88	84(86^a^)	80	88 (91^a^)
**3 markers**: **(90 % accuracy)** C5a (high) and/or Gliomedin (high) and/or TGFβ1 (high)	94	84(86^a^)	80	88 (91^a^)
**3 markers**: C5a (high) and/or Gliomedin (high) and/or Gal3ST1 (low)	92	84(86^a^)	80	88 (91^a^)
**4 markers**: **(92 % accuracy)** C5a (high) and/or Gliomedin (high) and/or TGFβ1 (high) and/or Gal3ST1 (low)	98 (b)	84(86^a^)	80	88 (91^a^)

**5 markers (94 % accuracy)**: C5a (high) and/or Gliomedin (high) and/or Gal3ST1 (low) and/or Interferon lambda-1 (low) and/or GHRH (high)	98 (b)	90	80	100

**6 markers**: C5a (high) and/or Gliomedin (high) and/or TGFβ1 (high) and/or Gal3ST1 (low) and/or Interferon lambda-1 (low) and/or LFA-3 (high)	98 (b)	92.5	85	100
**7 markers**: C5a (high) and/or Gliomedin (high) and/or TGFβ1 (high) and/or Gal3ST1 (low) and/or Interferon lambda-1 (low) and/or FASLG (high) and/or Transgelin (high)	98 (b)	97.5	95	100
9^c^**markers**:(**99**–**100 % accuracy)** C5a (high) and/or Gliomedin (high) and/or TGFβ1 (high) and/or Gal3ST1 (low) and/or Interferon lambda-1 (low) and/or GHRH (high) and/or GPNMB (high) and/or IGHG1 (low)	98 (100^b^)	100	100	100

A total of 11 markers are thus used in Table 7, consisting of C5a, Gliomedin and the other markers underlined consecutively in each box. A total of 4 targets are used for NP sensitivity, 2 additional targets for specificity versus HC, and 5 more targets for specificity vs CC controls.

a 91 % specificity with 1/24 subjects in the HC group being an extreme outlier C5a with 5 times the normal levels of C5a in HC, and 2X the high levels in NP can be differentiated providing 38/44, 86 % average specificity versus CC + HC controls using 2 biomarkers.

b 100 % with 1/48 NP subjects presenting as a super outlier Gal3ST1 for NEURO-PASC, at the normal-high Gal3ST1 level, plus normal-lowest TGFβ1, normal-lowest GHRH, normal-lowest FASL G, normal-high level of IFNλ1.

c Beyond these 9 markers in this box another two markers LFA-3 and Transgelin used for specificity versus CC are previously used in the rows above for specificity versus HC.

The last NP subject presented with very high GAL3ST1, as a super-outlier for NP on GAL3ST1 with levels at the upper level of normal versus controls, but with the lowest TGF-β1 (as well as lowest GHRH, and lowest FASLG levels in NP (and normal-high level of IFNλ1) described below as specificity differentiators. A more conservative sensitivity of 98 % is thus used in Table 7 above first broken line though it might be possible to get 100 % sensitivity∗∗.

For improved specificity to differentiate the remaining 3 HC subjects with high C5a (1 also with high Gliomedin) from NP subjects, 2 more biomarkers, normal levels of Interferon lambda-1 (IFNλ1) and Somatoliberin (GHRH) levels could be used, increasing the test specificity to 96 % (23/24) for HC; the other 1 HC subject differentiated as an extreme outlier, with extremely high C5a levels 5 times above HC samples and twice the levels of a NP subject taking it to 100 % specificity vs HC. Table 7 (see between the first broken line and second broken line). This provided an average 90 % specificity with the controls, HC (100 %) and CC (80 %), and with a sensitivity of 98 % and an accuracy of 94 % with 5 markers (Table 7).

For improved specificity to differentiate the remaining 4 CC subjects with high C5a and/or Gliomedin (Fig. 1b) from NP subjects, normal levels of 5 more biomarkers LFA-3, FASLG/Transgelin-1, and GPNMG/IgGH1, on top of the 2 IFNλ1 and GHRH used in the specificity differentiation of HC versus NP could be used (Table 7, see below the second broken line in the table).

Differentiating 1 CC subject involved 2 biomarkers, IFNλ1 and LFA-3. Differentiating this CC subject provided 17/20 85 % specificity versus CC and average 92.5 % specificity versus combined CC + HC groups.

Differentiating 2 more CC subjects required 2 more biomarkers, normal levels of FASLG and Transgelin 1 in combination with either normal IFNλ1 which provided 95 % specificity versus CC and up to 97.5 % specificity versus the combined CC + HC controls.

Differentiating the last CC subject required inclusion of 2 more biomarkers, normal levels of GPNMB and IgGH1 providing 100 % specificity for the CC, and the combined CC + HC controls.

Thus, using the 3 key biomarkers, high C5a, Gliomedin, and TGF-β1 provided 94 % sensitivity and it was possible to get 86 % sensitivity, providing 90 % accuracy and with low GAL3ST1 improving sensitivity (98 %) it was possible to get 92 % accuracy, and 2 additional markers IFNλ1 low and GHRH high versus HC provided 90 % specificity vs CC + HC, and 94 % accuracy. Together with 5 more specificity markers versus CC, it was possible to achieve close to 100 % sensitivity and 100 % specificity or 99 % accuracy (Table 7). Box plots of the 11 biomarkers for the diagnosis of NP subjects compared to CC and HC controls are in (**Supplementary Figure**) and in the drawings of published international patent application WO/2024/036373 (Tachas and Padhye).

### 3.13 C5a, gliomedin, and TGF-β1 diagnostic and therapeutic targets for subjects with Brain fog and/or fatigue

46 of 48 NP patients (2 were non-responders) reported their symptoms as reviewed in the methods. The subset of 39/46 (84.8 %) NP patients with the neurological symptom brain fog and 41/46 (89.1 %) with the non-neurologic symptom fatigue (Graham et al., 2021; Hanson et al., 2023; Visvabharathy et al., 2023) were assessed with the 3 primary diagnostic markers C5a, Gliomedin and TGFb1, and including with a fourth GAL3ST1. The subset of 37/46 subjects reporting both brain fog and fatigue were also assessed with these 3 primary diagnostic targets, and GAL3ST1 as a fourth.

The NP patients reporting brain fog and/or fatigue were detected as summarized below using the first three biomarkers at rates of 92–93 % and with the fourth marker at rates of 95–97 %, as follows.

• 39/46 (84.8 %) Subjects Brain fog
  • 36/39 (92 %) Subjects detected by 3 biomarkers
  • 38/39 (97 %) Subjects detected by 4 biomarkers
• 41/46 (89.1 %) Subjects did report symptoms of Fatigue
  • 38/41 (93 %) Subjects detected by 3 biomarkers
  • 40/41 (95 %) Subjects detected by 4 biomarkers
• 37/46 (80.4 %) subjects reported both Brain fog and Fatigue
  • 34/37 (92 %) Subjects detected by 3 biomarkers
  • 36/37 (97 %) Subjects detected by 4 biomarkers

**Fig. 3:**
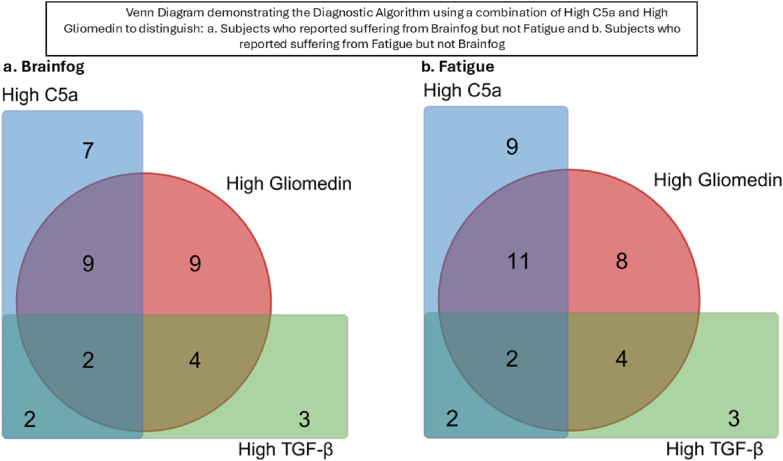
Venn Diagram demonstrating the Diagnostic Algorithm using a combination of High C5a and High Gliomedin to distinguish: a. Subjects who reported suffering from Brainfog but not Fatigue and b. Subjects who reported suffering from Fatigue but not Brainfog.

Of the (39/46) NP subjects assessed with brain fog, 36 were detected with the 3 markers including high TGF-β1 as a feature in 11 of these patients, 16 with only high C5a and 9 with only high Gliomedin (Fig. 3a)

Of the (41/46) NP subjects assessed with fatigue, 38 were detected with 3 markers, including high TGF-β1 as a feature in 11 of these patients, 20 only high C5a and 8 only high Gliomedin (Fig. 3b).

Statistical analysis was done with the 46 patients excluding 7 subjects for Brain fog and 5 subjects for fatigue. T-tests and U-tests comparing NP patients with brain fog (39) or fatigue (41) or both brain fog and fatigue (37) versus the combined CC + HC control groups identified all 3 targets at FDR <0.02 in the brain fog and fatigue groups, with Gliomedin at FDR <0.002 and at Bonferroni of <0.05 in both the U- and the T-tests.

ANOVA similarly identified the TGF-β1 target at FDR <0.02 for both brain fog (0.015) and fatigue (0.013), Gliomedin at FDR at <0.002 for brain fog (<0.001) and fatigue (0.002), and the C5a target at FDR <0.011 with fatigue, but with FDR = 0.037 for brain fog.

For subjects that had no brain fog but had other neurological symptoms, from headache, dizziness, dysgeusia, and anosmia, myalgia, numbness/tingling, or pain other than in the chest, high C5a (in 100 %) and high Gliomedin (in 50 %) appeared to be more closely associated in these subjects, in contrast to TGF-β1 which was normal in any of such subjects (Table 8).

**Table 8:** List of non-brain fog, non-fatigue reporters’ levels of C5a, Gliomedin and TGFβ1 biomarkers.

Group	Level	Target		
		C5a	Gliomedin	TGFβ1
No Brain Fog	High	4	2	0
	Normal	0	2	4
No Brain Fog/Fatigue	High	2	3	1
	Normal	1	0	2
No Fatigue	High	0	2	1
	Normal	2	0	1

In subjects with other non-neurologic symptoms, that is no fatigue, but depression and/or anxiety, and insomnia all had high gliomedin, a peripheral nerve target, normal C5a and a mix of high and normal TGF-β1.

## 4 Discussion

There is a socio-economic need to diagnose and treat individuals with Long COVID which is highly disruptive to both individuals and society. Vaccination and antivirals that target SARS-CoV-2 whilst reducing the severity of COVID-19 and incidence of Long COVID is not the answer to treatment of Long COVID, as recently exemplified by the failure of Paxlovid (Geng et al., 2024). The risk of Long COVID increases with more infections which will occur as the SARS-CoV-2 changes naturally over time and becomes more infectious and less virulent (Strong, 2022), with less vigilance on vaccination and milder COVID-19 symptoms. A significant number of Long COVID patients experience persistent symptoms for years including post mild COVID and symptoms like brain fog and fatigue can be long lasting and debilitating.

A range of mechanisms are reported to be the underlying cause of Long COVID including viral persistence in the body for up to 2 years, disruption to immune function, and microscopic blood clotting found in people in Long COVID trials (Paradiso et al., 2025; Saxena and Mautner, 2025; Peluso et al., 2024; Livingston et al., 2024). Studies that have only looked at a limited number of cytokines, have not identified markers that line up with the diagnostic targets identified herein, and provide no established criteria for diagnosis of Neuro-PASC. Non-pharmacological interventions are reviewed in Frontera et al. as having very low-to low-conditional support for improving pathological symptoms treatments (Frontera et al., 2023; Kwon et al., 2025; Robineau et al., 2025; Saxena and Mautner, 2025) and recently literature is described as scarce regarding the benefits of treatments (Whitaker-Hardin et al., 2025).

The present study uses the agnostic approach of proteomics to assess 7000 targets to determine targets modulated as potential diagnostic targets and targets for the potential treatment of Neuro-PASC compared to CC and/or HC control subjects. Using similar assessments, we previously discovered targets relevant to the Neuro-PASC pathological mechanisms and reported that Gliomedin and FasLG are related to brain fog (Hanson et al., 2023; Visvabharathy et al., 2023). The present study extends those findings and identifies the Gliomedin biomarker when combined with C5a and/or TGF-β1 have potential to diagnose Neuro-PASC subjects with 94 % sensitivity and up to 91 % specificity, in the subset of subjects with brain fog 92 % sensitivity, and in the subjects with fatigue 93 % sensitivity. C5a was interestingly also highly associated with non-brain fog neurological symptoms Neuro-PASC subjects. A more accurate diagnosis of all Neuro-PASC subjects was made with the addition of 1 more target GAL3ST providing 100 % sensitivity, and 2 more targets providing 100 % specificity versus HC controls, and 5 new targets providing 100 % specificity vs CC. Thus, beyond the 3 main targets 8 additional targets provided virtually 100 % sensitivity and specificity. One of these targets FasLG used in the present study for specificity versus CC subjects, was previously identified as associated with brain fog, suggesting it is possible to use both targets for sensitivity and specificity to characterize the pathology in Neuro-PASC.

The 11 diagnostic markers in the present study suggest neuroprotective-degenerative, viral-pathogen, vascular-adipose, clotting-platelet, autoimmune (inflammatory, fibrosis, integrin, antigen presentation) pathways are involved in Neuro-PASC pathogenesis and fatigue.

A further dozen partly overlying biomarkers were identified as modulated in Neuro-PASC and fatigue to which there are FDA approved drugs. These further markers were similarly involved in the processes identified using the diagnostic markers for which six biomarkers have FDA approved drugs or treatments in phase III trials.

The present findings suggest avenues to identify sub-groups of Neuro-PASC patients and diagnostic and therapeutic possibilities for those with fatigue with existing therapies to targets, as well as therapies at advanced stage of development (Tachas et al., 2025)

### 4.1 Convalescent and healthy controls patients compared to each other and Neuro-PASC

Convalescent subjects who had recovered 6 weeks past an infection or healthy subjects naive to SARS-CoV-2 infection had significantly different baseline levels of various immune biomarkers in their blood compared to subjects who had ongoing neurological PASC symptoms. There were no statistically significant differences in biomarkers expressed between the Convalescent Control and Healthy control group. On top of the 4 targets used for sensitivity to detect Neuro PASC subjects versus both CC and HC subjects, 2 more targets are needed to differentiate Neuro-PASC vs HC, and an additional 5 targets were needed to differentiate Neuro-PASC subjects versus CC specificity wise. One of the 5 specificity targets IGHG1 used towards 100 % Neuro-PASC specificity versus the CC, is related to a previously reported differentially expressed protein between Convalescent controls and Healthy controls, IgHV1 (Pushalkar et al., 2024). IGHG1 (uniprot P01857) in the present study is the IgG1 heavy constant region 1. In contrast IgHV1 is the variable region that participates in antigen recognition. IgG antibodies consist of 2 heavy chains, with 4 domains, one of which is the variable heavy chain which domain follows the constant heavy region 1.

### 4.2 Proteins C5 anaphylatoxin and gliomedin that serve as diagnostic biomarkers to identify Neuro-PASC subjects with 88 % sensitivity and 88–91 % specificity versus healthy controls and 80 % versus convalescent controls

C5a is a small peptide generated during the activation of the complement system, in response to physical or chemical damage. Upon cleavage of C5, C5a exerts its effects by binding to its receptors, C5aR1 and C5aR2, expressed on various immune cells (Guo and Ward, 2005; Monk et al., 2007). The binding of C5a to its receptors triggers a cascade of pro-inflammatory responses, including chemotaxis, degranulation, and cytokine production. Plasma C5a is elevated in neuropsychiatric SLE, associated with structural BBB integrity modulation and potentially cognitive dysfunction in SLE with elevated plasma neurofilament light chain indicating damage to the CNS (Sakuma et al., 2017; Kello et al., 2019; Zervides et al., 2022). Patients hospitalized with severe COVID-19 infection had higher levels of serum C5a and inhibition of C5a dampened inflammation associated with COVID-19 (Senent et al., 2021). C5a inhibitors such as Zilucoplan (De Leeuw et al., 2022) and Vilobelimab (NIH, 2019) have been used to treat hospitalized patients with severe COVID-19 infections.

GLDN is a protein that plays a crucial role in the formation and maintenance of the nodes of Ranvier in the peripheral nervous system. Nodes of Ranvier are essential for the rapid conduction of action potentials along myelinated axons (Han and Kursula, 2015). In the context of Neuro-PASC, there is growing evidence of neuroinflammatory processes and damage to the peripheral nervous system (Nalbandian et al., 2021; Moghimi et al., 2021). Elevated levels of GLDN could potentially indicate attempts to repair and restore damaged peripheral nerves. Studies have shown that neuroinflammation can lead to alterations in the expression of neuroprotective proteins including GLDN, with IgG anti-bodies to GLDN found in the sera of patients with multifocal motor neuropathy (Notturno et al., 2014). Auto antibodies to GLDN have been detected in other chronic demyelinating conditions such as multiple sclerosis (MS) involving the CNS and in particular chronic inflammatory demyelinating polyradiculoneuropathy involving the peripheral nervous system (Kira et al., 2019).

### 4.3 Proteins TGF-β1 and GAL3ST1 that in combination with C5a and gliomedin serve as diagnostic biomarkers for Neuro-PASC with 98 % sensitivity

TGF-β1 is a multifunctional cytokine that plays a critical role in regulating immune responses, tissue repair, and fibrosis (Sanjabi et al., 2017). Elevated levels of TGF-β1 have been associated with various inflammatory and fibrotic conditions, including viral infections and SARS-CoV-2 spike protein is reported to trigger barrier dysfunction and vascular leak via integrins and TGF-β1 signaling (Tresoldi et al., 2020), independent of ACE2 receptor. In the case of Neuro-PASC, where patients experience prolonged neurological symptoms post-acute infection, elevated levels of TGF-β1 could indicate ongoing immune dysregulation and tissue remodeling processes in the central or peripheral nervous system. Deregulation of TGF-β1 has been proposed for multiple neurological disorders including AIDS dementia complex, Alzheimer's disease, Parkinson's disease, Huntington's disease, amyotrophic lateral sclerosis (ALS), MS, anxiety, depression, and schizophrenia (Kashima and Hata, 2018). Pirfenidone is an anti-fibrotic drug that can prevent lung injury during SARS-CoV-2 infection by blocking the maturation process of TGF-β1 (Hamidi et al., 2021). The TGF-β1 signalling pathway, has been proposed as a potential therapeutic target to treat the Neuro-PASC symptoms in the chronic phase based on its immune suppressing effect (Oronsky et al., 2023). TGF-β1 has the potential to dampen inflammation and immune suppression can reduce the ability of the body to eradicate the virus consistent with recent reports of the virus presence in Long Covid (Oronsky et al., 2023). TGF-β inhibitors have not been successfully used to treat hospitalized patients with severe COVID-19 infections where TGF-β1 is increased though Pirfenidone works mostly in the lung (Oronsky et al., 2023). The use of Pirfenidone to treat Neuro-PASC is thus unclear.

The antisense drug ATL1102 to integrin CD49d works in the autoimmune disease MS patients to reduce inflammatory brain lesions, and in a phase 2a trial in Duchenne Muscular Dystrophy (DMD) patients modulates plasma molecules involved in reducing TGF-β1 activity (Limmroth et al., 2014; Woodcock et al., 2024). The CD49d, a4 chain of the VLA-4 (a4b1) adhesion molecule is a receptor for Rotavirus and murine polyomavirus, and a4b7 is the receptor for HIV-1, and b1 integrin, a receptor of other viruses (Tresoldi et al., 2020; Sigrist et al., 2020). VLA-4 is the hypothetical secondary receptor for SARS-CoV-2 beyond ACE2 receptor, which is relevant to Long COVID, with reports of a SARS-CoV-2 reservoir, and integrins involved in vascular leaks via TGF-β1 (Biering et al., 2022), thus potentially relevant to the CNS in brain fog. It awaits to be seen whether anti-viral, anti-inflammatory drugs like Bucillamine being trialed in Long COVID or BC 007 Rovunamptin, an aptamer drug to neutralize pathogenic functional autoantibodies (Haberland and Müller, 2022) also work in some Neuro-PASC or fatigue patients. Targeting integrin receptors and associated TGF-β1 activity may have potential benefit on viral, inflammatory, and fibrotic mechanisms in some patients with Long COVID.

Galactosylceramide sulfotransferase (GAL3ST1) is an enzyme involved in the biosynthesis of sulfatide, a sulfated glycosphingolipid found in the myelin sheath of neurons. Sulfatide, synthesized by GAL3ST1, is an essential component of myelin and plays a crucial role in maintaining the structural integrity and function of neuronal membranes. Low levels of GAL3ST1 could indicate reduced biosynthesis of sulfatide in response to neuronal damage or demyelination associated with neuroinflammation. Studies have shown that alterations in sulfatide metabolism, including changes in GAL3ST1 expression, are linked to neurodegenerative diseases and demyelinating disorders. Sulfatide has been implicated in modulating immune responses and neuroinflammation in the central nervous system. Low levels of GAL3ST1 and sulfatide could reflect ongoing dysregulation of inflammatory processes and neuronal damage in patients with Neuro-PASC.

### 4.4 Proteins IFNλ1 and GHRH that in combination with C5a and gliomedin diagnostic biomarkers differentiated Neuro-PASC subjects from healthy subjects with 100 % specificity

IFNλ1 is a type III interferon, which plays a crucial role in the antiviral defense mechanism (Misumi and Whitmire, 2014). IFNλ1 is reported elevated in Long COVID-19 subjects versus healthy controls at 4 and 8 months post mild-moderate SARS-CoV-2 infection (Phetsouphanh et al., 2022). The Neuro-PASC patients in the present study 3–9 months following acute COVID-19 however, have 29 % downregulation of IFNλ vs HC suggesting differences in INFλ signatures in the present Neuro-PASC study patients. Studies have demonstrated that IFNλR deficient mice had a diminished T-cell response to persistent infection (Misumi and Whitmire, 2014). Pegylated IFNλ drugs in phase III development used as antiviral treatments, accelerate SARS-CoV-2 clearance in nasopharyngeal swabs, with further studies suggesting treatment is more effective in individuals with high viral load, administered early, whilst another study showed contradictory results (Interferon Lambda). The present Neuro-PASC study suggests low levels of IFNλ1 may need to be considered when treating subjects with Long COVID with IFNλ1.

Somatoliberin (GHRH) is primarily produced in the hypothalamus and has a primary role in regulating the release of Growth Hormone (GH) from the pituitary gland which then leads to the production of another growth hormone Insulin-like Growth Factor 1 (IGF-I). GHRH has been shown to have neuroprotective and neurotrophic effects in the brain, as has GH and IGF-I. Studies have suggested that dysregulation of GHRH signaling, leading to elevated levels of GHRH, may however, contribute to neuroinflammation through Th17 cell-mediated autoimmune inflammation (Du et al., 2023). It is possible the body in seeking to improve neural function via increasing GHRH there is modulation of TH17 inflammation as low GH secretion is associated with Neuro-PASC (Wright et al., 2024a). GH treatment, however, improved scores for fatigue but not cognition in a recent completed clinical study (Wright et al., 2024b).

### 4.5 Proteins LFA-3, FASLG + Transgelin, and GPNMG + IgGH1 in combination with C5a, gliomedin, IFNλ1 and GHRH that serve as diagnostic biomarkers to differentiate Neuro-PASC subjects from convalescent and healthy control subjects with 100 % specificity

Lymphocyte-function antigen 3 (LFA-3), also known as CD58 glycoprotein, is a receptor primarily activated by its ligand, CD2, which is expressed naturally by T-cells and NK cells. The CD58^−^CD2 interaction promotes cell-cell adhesion that plays a role in T-cell activation and signaling, as well as proliferation and cytokine production. Upregulation of adhesion biomarkers such as CD58 was observed in patients with chronic fatigue syndrome (CFS) and suggests it may arise as a consequence of antigen exposure (Straus et al., 1993). Increases in IL17a expression seen post autologous haematopoietic stem cell transplantation (aHSCT) was partially attributed to CD58, through its co-stimulation of Th17 cells (Miossec and Kolls, 2012). In the same study, the NK cells also caused the same Th17 cells to undergo NKG2D-dependent cytotoxicity, killing them in an attempt to maintain immunological memory while preventing aberrant T-cell signaling.

Tumor necrosis factor ligand superfamily member 6 (FASLG) or Fas-Ligand is type II membrane protein belonging to the tumor necrosis factor (TNF) 1 family of proteins and is the ligand for the Fas receptor/CD95 (Nagata, 1997; Schneider et al., 1997). FASLG is involved in cytotoxic T-cell-mediated apoptosis, NK cell-mediated apoptosis and in T-cell development (Alderson, 1995). NP subjects had an elevated level of FASLG, suggesting an aberrant immune environment.

TAGLN also known as SM22α, is a smooth muscle marker and a tumor suppressor, a member of the calponin family of actin-binding proteins (Matsui et al., 2018; Liu et al., 2020). While other members of the family have been associated with diseases such as asthma (Yin et al., 2018), TAGLN is more commonly associated with Colorectal Cancer, with expression being elevated in patients with advanced disease. TAGLN is involved downstream of TGF-β1, TNF, EGFR, and other growth factor PI3k pathways, which lead to migration invasion.

### 4.6 Neuro-PASC subjects with Brain fog have either high TGF-β1 and/or high C5a, and/or high gliomedin

The prevalence of brain fog in COVID patients not requiring hospitalization is 80 %, versus 86 % post-hospitalization (Graham et al., 2021). Though hospitalized patients may perform overall worse in cognitive tests, non-hospitalized patients perform 10 % worse than healthy subjects in established cognitive tests including attention deficits (Graham et al., 2021). Trouble with memory or focussing was the second most common symptom of 18 prolonged symptoms in school-age children 6–11 years of age (44 %) and in adolescents, of 17 symptoms (47 %) (Gross et al., 2024). Persistent cognitive impairment lasting up to 12 months, reported in 26 % of subjects 50–65 % of COVID-19 who required hospitalization (Gross et al., 2024). Particularly disruptive to individuals and society are Long Covid “long-haulers” who have been unwell for years with neurological pathological symptoms of brain fog and fatigue with unknown cause. Cognitive symptoms are the long lasting ones reported in 26 % of subjects at 12 months (Cysique et al., 2022), and at 2 yrs occurs in children (3.9 %), adults (6.4 %), and elderly (15.4) with 16.7 % of affected adults and 61 % of elderly having died at 2 yrs (Taquet et al., 2022).

In the present study a subset of the 11/39 Neuro-PASC subjects with brain fog were identified with high TGF-β1. Interestingly SPARC is reduced in Neuro-PASC vs CC at BF < 0.05, is similarly reduced in the blood post sports concussion, though it is expressed more highly in blood vessels involved in BBB angiogenesis where it inhibits DNA synthesis in TGFβ sensitive cells (Schiemann et al., 2003). SPARC interacts with thrombospondin-1 (Tsuda, 2018) which similarly inhibits DNA synthesis, and may activate TGF-β1. Reduction in NP subjects SPARC which blocks SPARCL1 is consistent with 114 % increase in SPARCL1 in NP vs HC and suggests these biomarkers may be associated with brain fog. There are limited associations between C5a and Gliomedin with SPARC so the remaining 25/39 subjects with high C5a and Neuro-PASC brain fog may have other proteins co-modulated.

The high TGF-β1, C5a diagnostic and Gliomedin levels together with clinical assessment could be used to personalize treatments in 92 % of Neuro-PASC patients with brain fog, fatigue, or both with existing TGF-β1 and C5a therapies and or therapies in late-stage development available for testing to determine the benefits.

### 4.7 Diagnostic biomarkers differentiate Neuro-PASC subjects from ME/CFS, and other conditions

There is an overlap in the Long COVID symptoms of fatigue, post-exertional malaise after physical or mental effort, and cognitive dysfunction (brain fog), with Myalgic Encephalomeylitis/Chronic Fatigue Syndrome (ME/CFS) symptoms. Interestingly, the TNFRSF1A gene encoded TNF sR-1 protein elevated 29 % in Neuro-PASC, is also 1.28-fold increased in ME/CFS patients in a proteomics study using the SomaScan® platform (Germain et al., 2021). In that study, however, TGF-β1 is slightly lower, differentiating ME/CFS from Neuro-PASC patients, 41/46 of whom had fatigue, and 39/46 brain fog.

The diagnostic targets identified for Neuro-PASC subjects are also distinguished from autoimmune disease such as MS. In MS it is reported EBV serology against nuclear antigen EBNA1 and capsid antigen VCA is necessary but not sufficient in the pathophysiology (Bose et al., 2024). Several studies have sought to similarly link EBV, or other herpesvirus infections, to PASC through serology and epidemiological differences in viremia, however more research is required to determine whether there is an association (Su et al., 2022).

The immune changes observed in the 38/46 Neuro-PASC subjects who reported Non-neurologic symptoms anxiety and/or depression, do not overlap with immune cell and the immune cell mediators modulated in clinically significant depression, or anxiety when excluding comorbid conditions (Kuring et al., 2023).

### 4.8 Diagnostic biomarkers altered in Neuro-PASC subjects to which there are existing therapeutics

Six of the eleven diagnostic biomarker proteins in the blood of Neuro-PASC subjects significantly altered from CC subjects who recovered from COVID-19 without Neuro-PASC and from HC subjects who were never infected with COVID-19 can be targeted with modulatory therapies. Twenty proteins in total were identified as modulated in the non-hospitalized Neuro-PASC subjects to which there are established modulatory therapies or therapies that are in Phase II or III Trials (Table 6). Many of these proteins involve immune and inflammatory responses with overlapping roles to the diagnostic biomarkers. Future research is required to understand how they may be involved in the underlying cause of the aberrant immune response to COVID-19 and/or infection that persists into the post-acute and chronic Long COVID phase.

C5a identified as 1 of the key diagnostic markers for sensitivity expressed more highly in the non-hospitalized Neuro-PASC subjects, with roles in modulating BBB, interestingly has been targeted successfully by antibody Vilobelimab in the treatment of different symptoms in patients with high levels of C5a hospitalized with severe COVID-19^45−47^ and is a registered therapy (Aboul-Fotouh et al., 2023). Intriguingly antihistamines used to treat symptoms attributed to mast cell activation in long COVID, reduced in 6 of 14 subjects self-reported brain fog (Whitaker-Hardin et al., 2025; Salvucci et al., 2023) and mast cells are activated via the C5aR (Ansotegui et al., 2022). Using C5a as a diagnostic and targeted therapeutic patient-centered intervention in subjects with brain fog is worth exploring.

Polypeptide, protein modulators such as Pegylated IFNλ with potential in clearing SARS-CoV-2 trials in Neuro-PASC patients with low IFNλ may be helpful to test the hypothesis that COVID-19 infection persisting in Long COVID is the cause of neurological symptoms. Peptide modulators like MR 409 (GHRH) is available to a second targets used in diagnostics for specificity. GHRH may be helpful neurologically or may be pro-inflammatory, and data from more trials will indicate its role in the group of subjects. GH treatment significantly improved neurologic symptoms in PASC patients but cognition, sleep, and physical performance were not significantly altered. A diagnostic and targeted therapeutic patient-centered intervention may be considered to assess if it changes outcomes.

Other drugs include small molecule modulators to two of the three key diagnostic targets TGF-β1 and C5a used in sensitivity. Pirfenidone targets TGF-β1 though mostly in the lung which may or may not be helpful in Neuro-PASC brain fog. Deupirfenidone, a form of pirfenidone is reported to have the anti-fibrotic anti-inflammatory activity of pirfenidone and is in phase II study NCT04652518 in post -acute COVID. Interestingly, there is cross talk reported between TGF-β1 and complement activation in pulmonary fibrosis, and future research is needed to determine if this may be related to the observation 4/36 subjects with brain fog had both elevated TGF-β1 and C5a. Ravilizumab targets Complement C5b-C6 complex and may be useful perhaps more active in the periphery than in the CNS, and its potential in the treatment of Neuro-PASC subjects can also be considered in future studies.

### 4.9 Limitations

The observations of nearly 100 % sensitivity and specificity using 11 biomarkers of the 7000 plasma proteins are based on a small dataset of 48 NP and 44 Control subjects and would benefit from external validation with a larger dataset. The use of C5a, TGFβ1, and Gliomedin, together to differentiate patients with Neuro-PASC from Control subjects at 94 % sensitivity and 86 % specificity needs to be validated. A similar size number of NP and Control subjects as used in the present study (n = 43 per group) can provide 80 % powering for 86 % specificity, and a larger size number of 160 subjects per group can provide 90 % powering for 80 % sensitivity or specificity and would be useful external validation datasets for diagnostics.

## 5 Conclusion

Through the comprehensive proteomic quantification of plasma samples, and approach to identify potential diagnostic biomarkers with high sensitivity and specificity and therapeutic targets, this study reveals important new insights into the altered neural, autoimmune, viral, and vascular environment of Neuro-PASC and the modulation of immune cells (Graham et al., 2021; Hanson et al., 2023; Visvabharathy et al., 2023). This novel approach may be used in further investigations of mechanisms in other PASC and disease.

Using the SomaScan® platform testing for 7000 plasma proteins, a few dozen plasma proteins were identified that were expressed differentially in the plasma of adult patients with Neural Post-acute sequelae of COVID-19 (Neuro-PASC) who had not been hospitalized by SARS-CoV-2, compared to both Convalescent Control and Healthy Control subjects prior to vaccination. These people were not anticipating Neuro-PASC.

Using 11 biomarkers in combination enabled differentiation of Neuro-PASC subjects from Healthy Control and Convalescent Control subjects with high sensitivity and high specificity. As few as 3 key biomarkers, known to be highly expressed in serum, high C5a, Gliomedin, and TGF-β1, provided 94 % sensitivity, 86 % specificity, and 90 % accuracy in the present study. Additional markers beyond immunomodulatory markers (C5a, TGFβ1) are neuroprotective neurodegenerative markers (GLDN, GAL3ST1) and inflammatory markers, LFA3, IFNλ1, and TNF-related proteins, and neurodegenerative-inflammatory markers GHRH.

The present study data supports examining existing and new Neuro-PASC plasma samples, and, testing a small number of antibodies using Elisa and other conventional pathology laboratory approaches, mass spectroscopy or proteomics. This study opens promising avenues of investigation for the development of diagnostic and targeted therapeutic patient-centered intervention in Neuro-PASC and associated post COVID fatigue.

## CRediT authorship contribution statement

**A.S. Padhye:** Writing – review & editing, Writing – original draft, Validation, Resources, Project administration, Investigation, Formal analysis, Data curation. **I.J. Koralnik:** Writing – review & editing, Validation, Supervision, Resources, Conceptualization. **B.A. Hanson:** Writing – review & editing, Validation, Supervision, Investigation, Data curation. **L. Visvabharathy:** Supervision, Resources, Project administration, Investigation. **R.K. DeLisle:** Supervision, Software, Resources, Investigation, Formal analysis, Data curation. **G. Tachas:** Writing – review & editing, Writing – original draft, Visualization, Validation, Supervision, Resources, Project administration, Methodology, Investigation, Funding acquisition, Formal analysis, Conceptualization.

## Declaration of competing interest

The authors declare the following financial interests/personal relationships which may be considered as potential competing interests: Dr Koralnik, Dr Hanson, as employees of Northwestern University and Dr Visvabharathy as an employee of the University of Chicago (at the time of the study an employee of Northwestern University) have no competing financial interest or relationships that could have influenced the work reported in this paper.Dr DeLisle, Glysade LLC, at the time of the study an employee of SomaLogic, Inc, Boulder, CO 80301, USA, have no competing financial interest or relationships that could have influenced the work reported in this paper.Mr Padhye and Dr Tachas at the time of the study, were employees of the sponsor Percheron Therapeutics Ltd (formerly Antisense Therapeutics Ltd), and received payment for services from the sponsor as employees. Mr Padhye was until January 31, 2025 an employee of the sponsor.Dr George Tachas was until March 7, 2025 an employee of the sponsor and holds an equity interest in the sponsor. Dr Tachas is the named inventor on the sponsor's patent application PCT/AU2023/050777 in relation to diagnosis and treatment of neuro-PASC, and in the process of in-licensing via an agreement with the sponsor.The author's interest does not influence the objectivity and integrity of work reported in this paper.

## Data Availability

Data will be made available on request.
