# Supplementary material for: Blood diagnostic biomarkers for neurologic manifestations of long COVID
Source: Brain Behav Immun Health. 2025 Sep 25;49:101110. doi: 10.1016/j.bbih.2025.101110 (PMC12554056; doi:10.1016/j.bbih.2025.101110)
Supplement: Multimedia component 5 [file mmc5.pdf]

Supplementary Figure 5:

Box plots of the Diagnostic Targets generated using Dataviz® software.
Y-axis Units are in RFu.

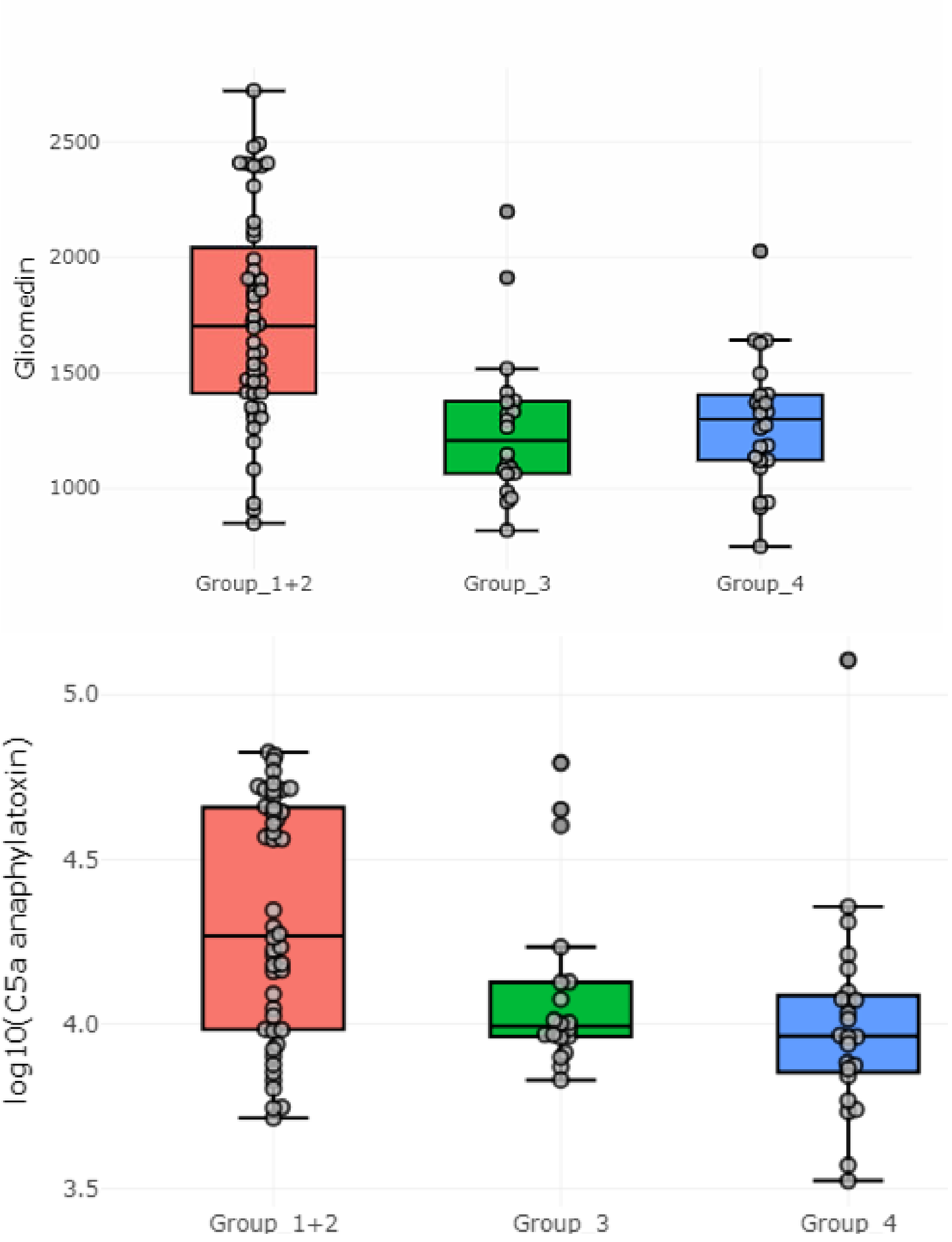

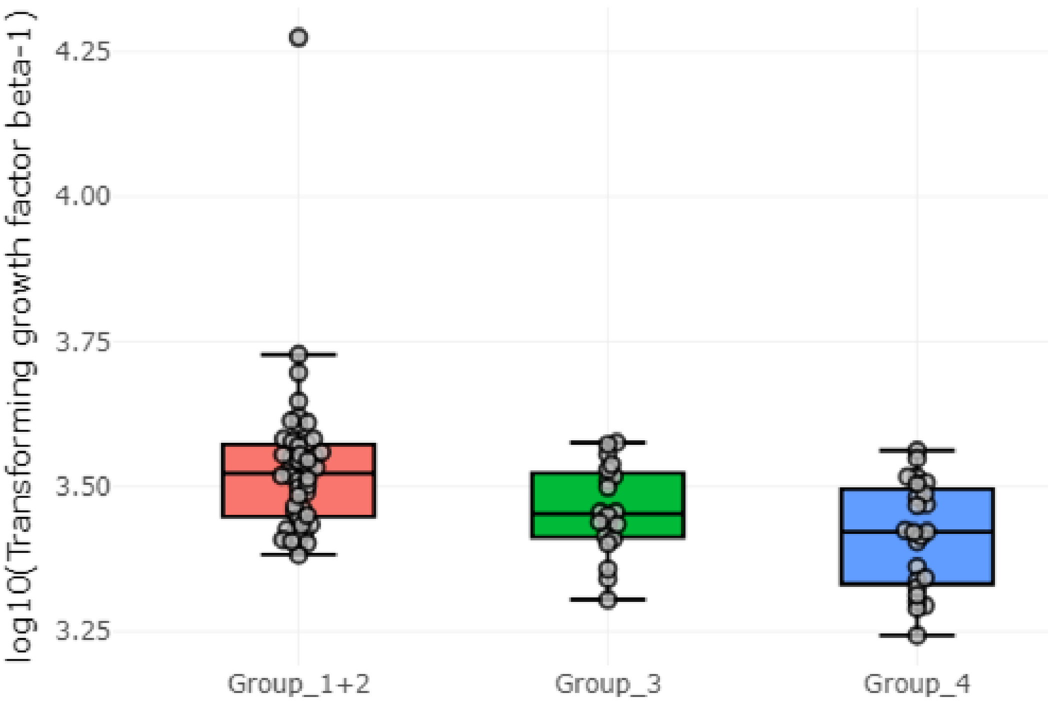

log10(Transforming growth factor beta-1)
4.25
4.00
3.75
3.50
3.25
Group_1+2
Group_3
Group_4

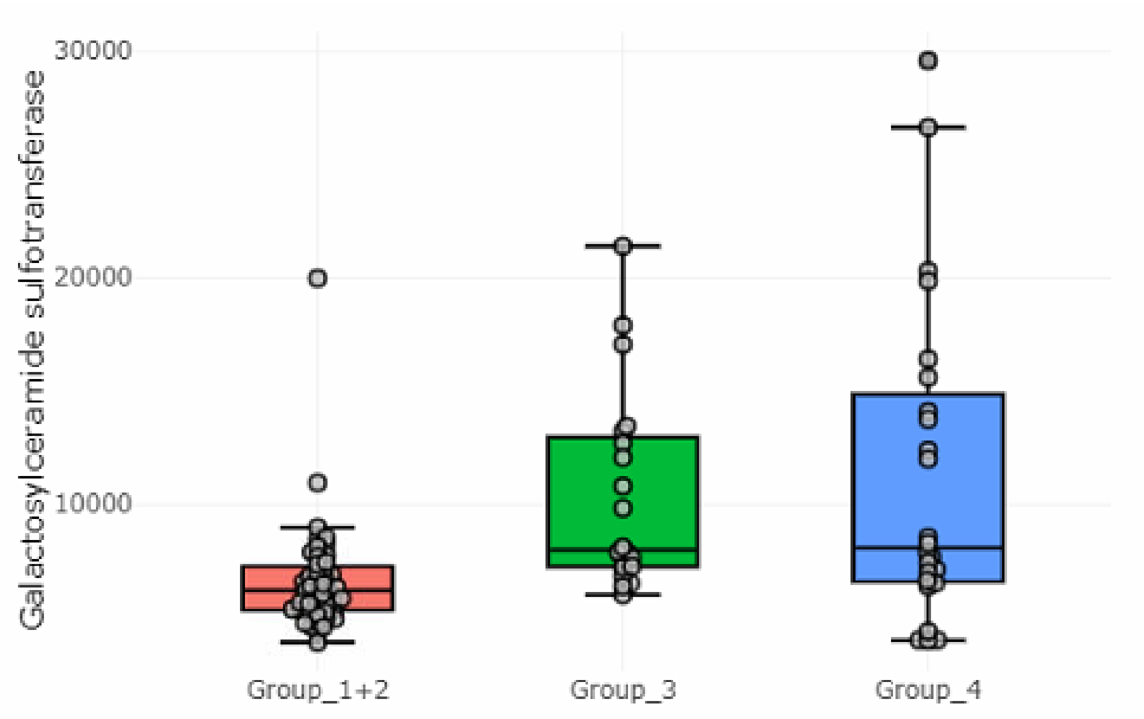

Galactosylceramide sulfotransferase
30000
20000
10000
Group_1+2
Group_3
Group_4

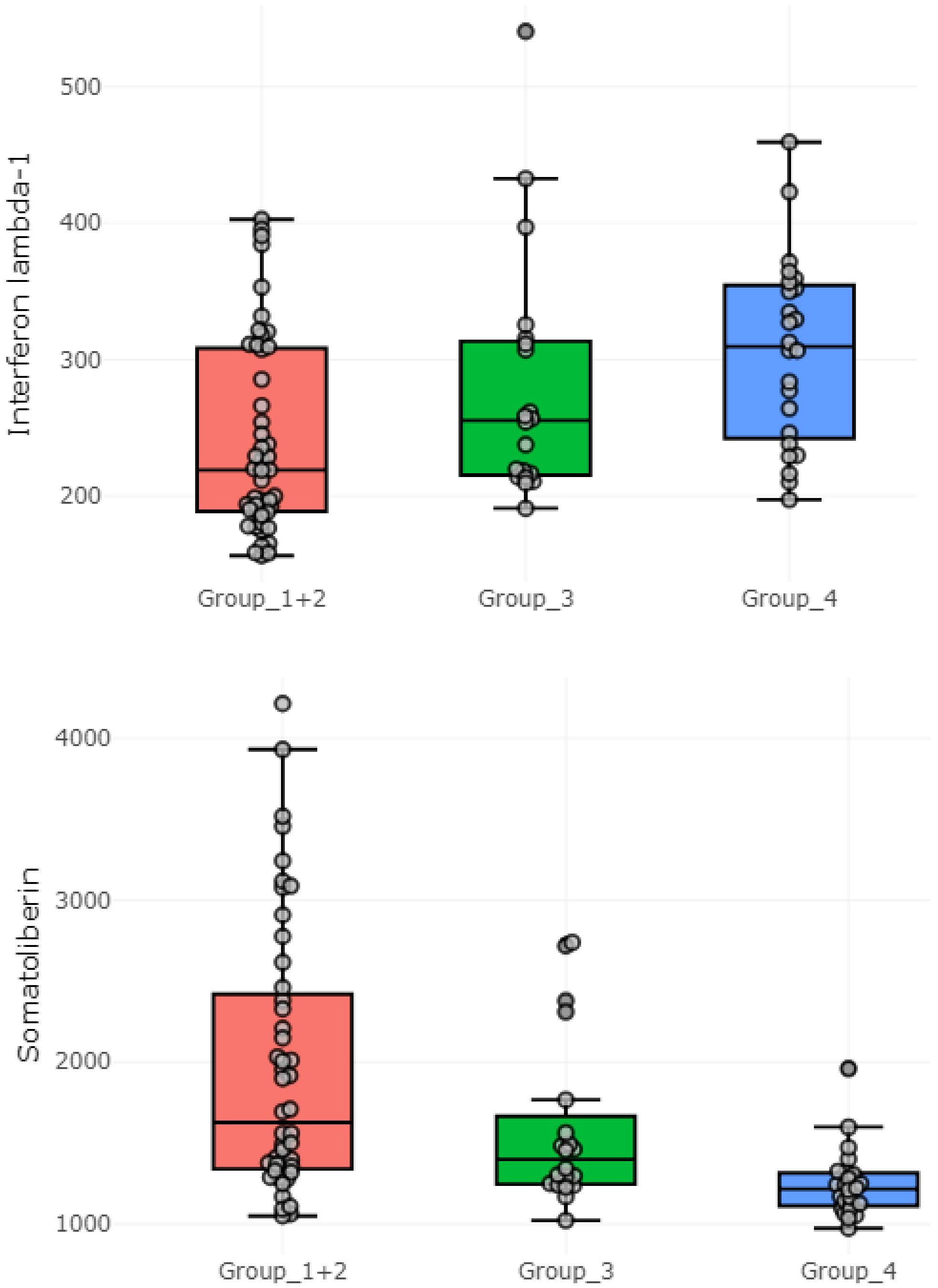

Interferon lambda-1
500
400
300
200
Group_1+2
Group_3
Group_4
Somatoliberin
4000
3000
2000
1000
Group_1+2
Group_3
Group_4

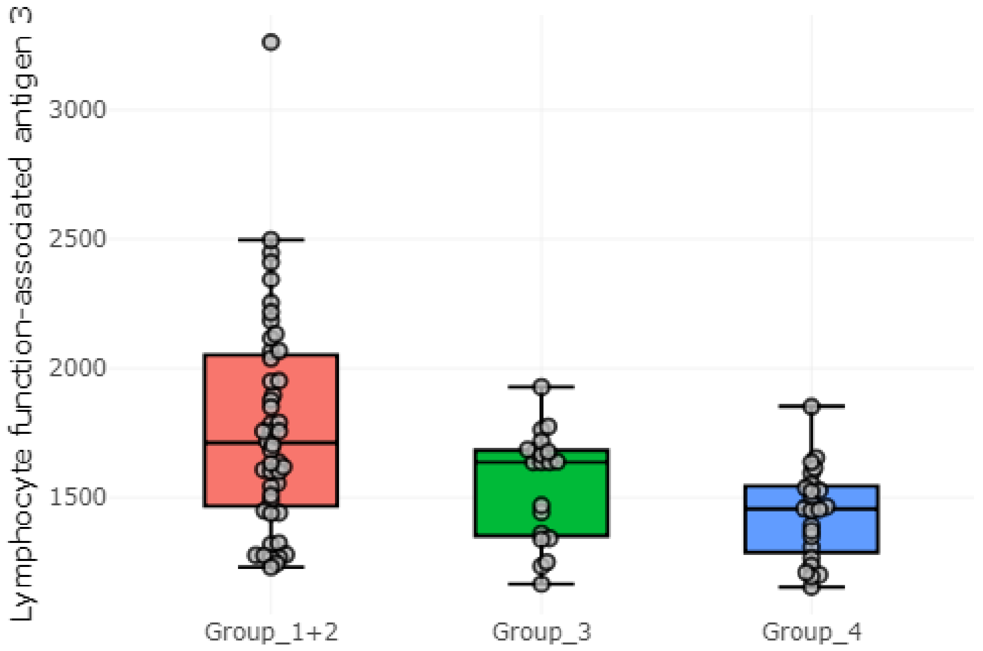
Lymphocyte function-associated antigen 3
3000
2500
2000
1500
Group_1+2
Group_3
Group_4

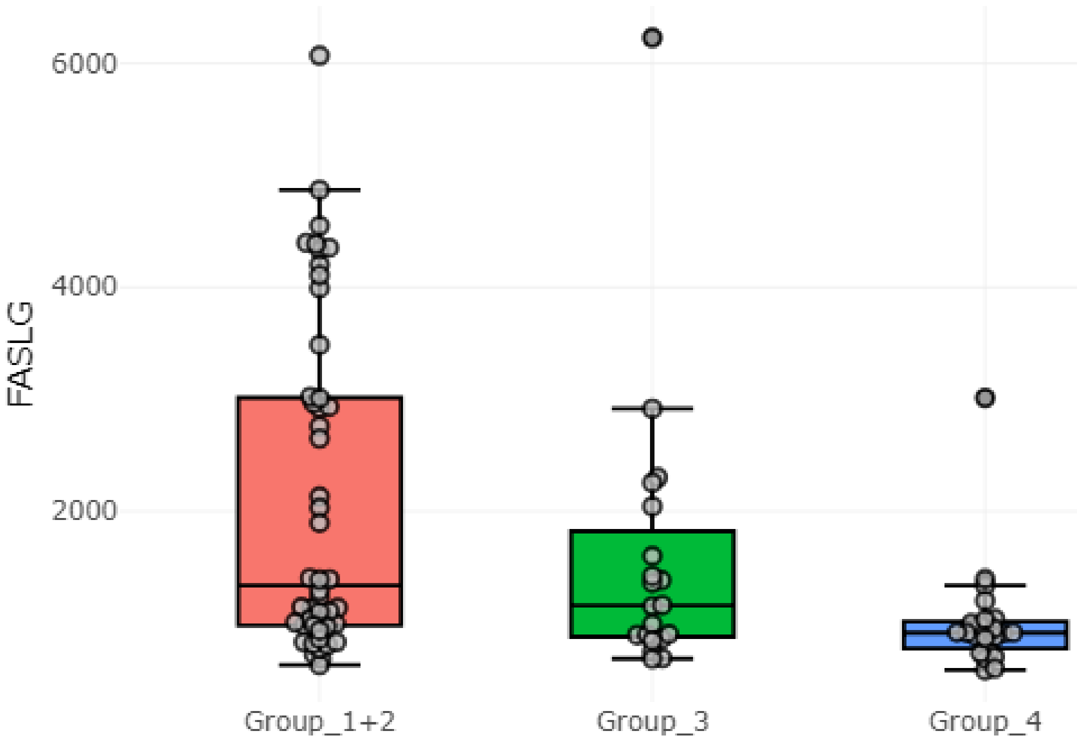
FASLG
6000
4000
2000
Group_1+2
Group_3
Group_4

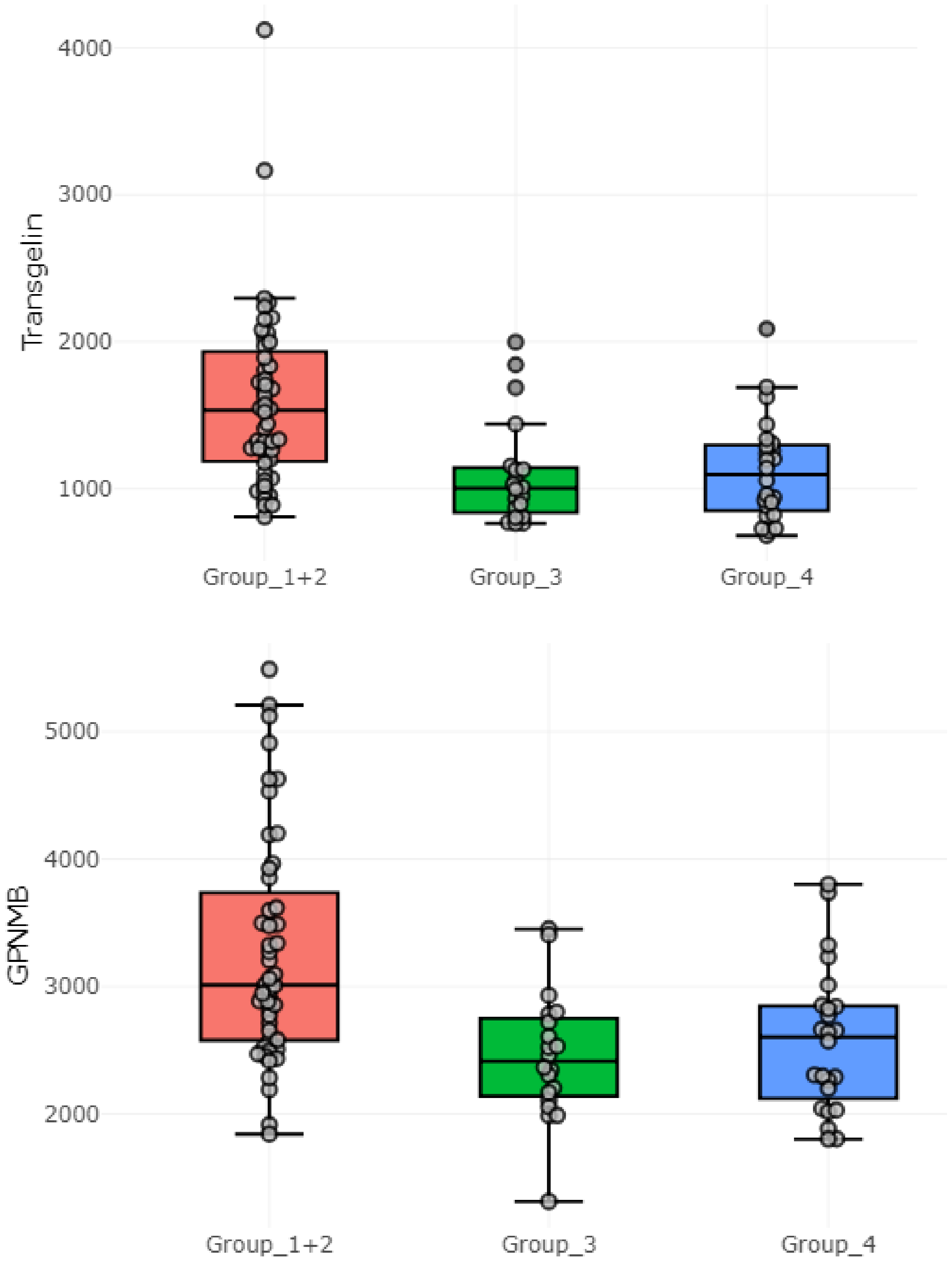
Transgelin
4000
3000
2000
1000
Group_1+2
Group_3
Group_4
GPNMB
5000
4000
3000
2000
Group_1+2
Group_3
Group_4

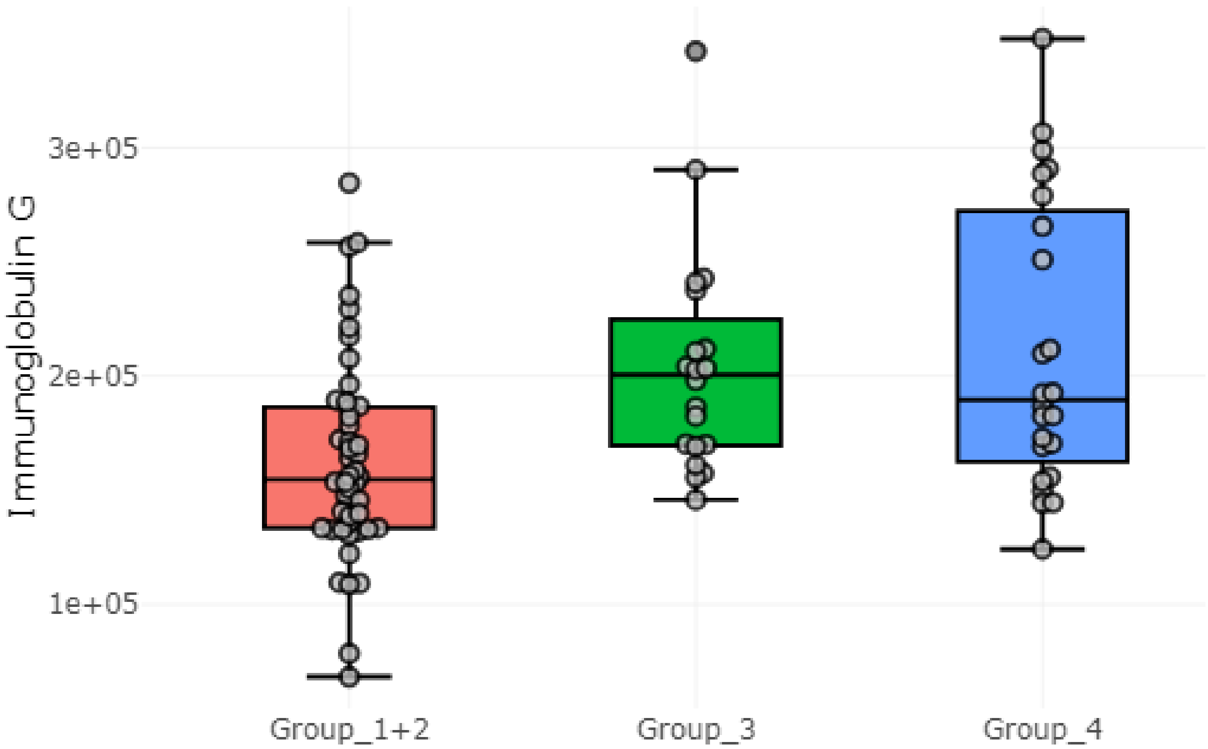

3e+05
2e+05
1e+05
Immunoglobulin G
Group_1+2
Group_3
Group_4
